# Outer membrane vesicles from *Neisseria gonorrhoeae* target PorB to mitochondria and induce apoptosis

**DOI:** 10.1371/journal.ppat.1006945

**Published:** 2018-03-30

**Authors:** Pankaj Deo, Seong H. Chow, Iain D. Hay, Oded Kleifeld, Adam Costin, Kirstin D. Elgass, Jhih-Hang Jiang, Georg Ramm, Kipros Gabriel, Gordon Dougan, Trevor Lithgow, Eva Heinz, Thomas Naderer

**Affiliations:** 1 Biomedicine Discovery Institute and Department of Biochemistry & Molecular Biology, Monash University, Clayton, Victoria, Australia; 2 Biomedicine Discovery Institute and Department of Microbiology, Monash University, Clayton, Victoria, Australia; 3 Monash Ramaciotti Centre for Cryo Electron Microscopy, Monash University, Clayton, Victoria, Australia; 4 Monash Micro Imaging, Monash University, Clayton, Victoria, Australia; 5 Infection Genomics Program, Wellcome Trust Sanger Institute, Hinxton, United Kingdom; Gifu University, JAPAN

## Abstract

*Neisseria gonorrhoeae* causes the sexually transmitted disease gonorrhoea by evading innate immunity. Colonizing the mucosa of the reproductive tract depends on the bacterial outer membrane porin, PorB, which is essential for ion and nutrient uptake. PorB is also targeted to host mitochondria and regulates apoptosis pathways to promote infections. How PorB traffics from the outer membrane of *N*. *gonorrhoeae* to mitochondria and whether it modulates innate immune cells, such as macrophages, remains unclear. Here, we show that *N*. *gonorrhoeae* secretes PorB via outer membrane vesicles (OMVs). Purified OMVs contained primarily outer membrane proteins including oligomeric PorB. The porin was targeted to mitochondria of macrophages after exposure to purified OMVs and wild type *N*. *gonorrhoeae*. This was associated with loss of mitochondrial membrane potential, release of cytochrome *c*, activation of apoptotic caspases and cell death in a time-dependent manner. Consistent with this, OMV-induced macrophage death was prevented with the pan-caspase inhibitor, Q-VD-PH. This shows that *N*. *gonorrhoeae* utilizes OMVs to target PorB to mitochondria and to induce apoptosis in macrophages, thus affecting innate immunity.

## Author summary

*Neisseria gonorrhoeae* causes the sexually transmitted disease gonorrhoea in more than 100 million people worldwide every year. The bacteria replicate in the reproductive tract by evading innate and adaptive immunity. In the absence of effective vaccines and the rise of antibiotic resistance, understanding the molecular interactions between innate immune cells and *N*. *gonorrhoeae* may lead to new strategies to combat bacterial growth and the symptoms of gonorrhoea. It has long been known that the *N*. *gonorrhoeae* porin, PorB, promotes bacterial survival but also targets host mitochondria in infections. The mechanism by which PorB traffics form the bacterial outer membrane to host mitochondria remains unclear. Here, we utilized proteomics and super-resolution microscopy to show that *N*. *gonorrhoeae* secretes PorB via outer membrane vesicles. These vesicles are taken up by macrophages and deliver PorB to mitochondria. Macrophages treated with *N*. *gonorrhoeae* vesicles contained damaged mitochondria and active caspase-3. A caspase inhibitor prevented apoptosis of macrophages treated with *N*. *gonorrhoeae* vesicles. This suggests that *N*. *gonorrhoeae* secretes membrane vesicles, which are readily detectable in gonorrhoea patients, to target macrophages and to promote infections.

## Introduction

*Neisseria gonorrhoeae* causes the sexually transmitted disease gonorrhoea. With more than 100 million cases reported every year, gonorrhoea is the second most commonly reported sexually transmitted bacterial infection. In addition, gonorrhoea promotes the transmission of HIV [1, 2]. *N*. *gonorrhoeae* replicates primarily extracellularly within the mucosa of reproductive organs, resulting in localized inflammation but also pelvic inflammatory diseases due to bacterial dissemination. Mucosal resident macrophages and the recruited neutrophils and monocytes fail to control *N*. *gonorrhoeae* replication [3]. It is thought that *N*. *gonorrhoeae* modulates the antimicrobial activities of neutrophils and macrophages, including apoptosis pathways, to promote bacterial survival [4]. Unlike many other pathogens, however, there is little evidence that *N*. *gonorrhoeae* utilizes secretions systems and secreted cytotoxins to target host cells [3]. Rather, the major porin protein, PorB, has been identified to modulate apoptotic pathways in *N*. *gonorrhoeae* infections [4–11].

PorB is expressed on the outer membrane of *N*. *gonorrhoeae* as a homotrimeric ß-barrel protein complex [11, 12]. The porin is a voltage gated pore that facilitates ion exchange and the uptake of small nutrients, essential for bacterial viability [13]. Expressed in mammalian cells, PorB from *N*. *gonorrhoeae* is recognized by the mitochondrial import machinery, partly because of the evolutionary conservation with the bacterial outer membrane assembly machinery [14]. The translocase of the outer mitochondrial membrane (TOM) recognizes unfolded PorB and the sorting and assembly machinery (SAM) together with small intermembrane space chaperones enable folding within the outer mitochondrial membrane [6, 9, 15]. Besides mitochondrial import, PorB is also thought to share functional similarities with the endogenous porin, voltage-dependent anion channel (VDAC), including the regulation of the mitochondrial membrane potential and apoptotic death factors [5, 6, 9]. PorB may also target the inner mitochondrial membrane to directly trigger loss of membrane potential regulated by ATP binding [9, 12, 16]. How the porin affects apoptosis, however, remains unresolved and controversial, as PorB from *N*. *gonorrhoeae* requires additional host factors to trigger apoptosis and PorB in *N*. *meningitidis* prevents mitochondria-mediated cell death signaling [10, 17, 18].

Little is known about how the homotrimeric ß-barrel PorB, containing 16 transmembrane spanning segments per monomer, traffics from the bacterial outer membrane to host mitochondria. PorB may directly translocate from *N*. *gonorrhoeae* outer membranes into the plasma membranes of host cells due to the close contact of bacteria and the epithelial layer via an uncharacterized mechanism [19, 20]. The major porins of *Pseudomonas aeruginosa*, *Vibrio cholera* and *Neisseria meningitidis* also translocate from the outer membrane to host mitochondria [17, 21, 22], suggesting that bacterial pathogens may use a common strategy for the delivery of membrane proteins to host organelles. Outer membrane vesicles (OMVs) are produced by essentially all Gram-negative bacteria and are increasingly recognized as a major delivery system in infections [23–27]. This includes bacterial toxins that are packaged as soluble molecules within the lumen of OMVs to target host cells [27]. OMVs also share proteins with the outer membrane including porins [26, 28, 29]. Whether OMVs enable trafficking of *Neisseria* porins to mitochondria and activate apoptosis remains unknown.

Here, we report that *N*. *gonorrhoeae* employs OMVs to interact with macrophages and to deliver PorB to the mitochondria. Highly purified OMVs from *N*. *gonorrhoeae* contain a core proteome of 110 proteins, of which PorB is the major protein. We provide evidence that PorB is trafficked as folded oligomeric complex in OMVs. Despite this, PorB translocates to mitochondria in OMV-treated cells and purified organelles, which was sufficient to cause the loss of mitochondrial membrane potential and macrophage apoptosis.

## Results

### Purification of *N*. *gonorrhoeae* OMVs

**Fig 1:**
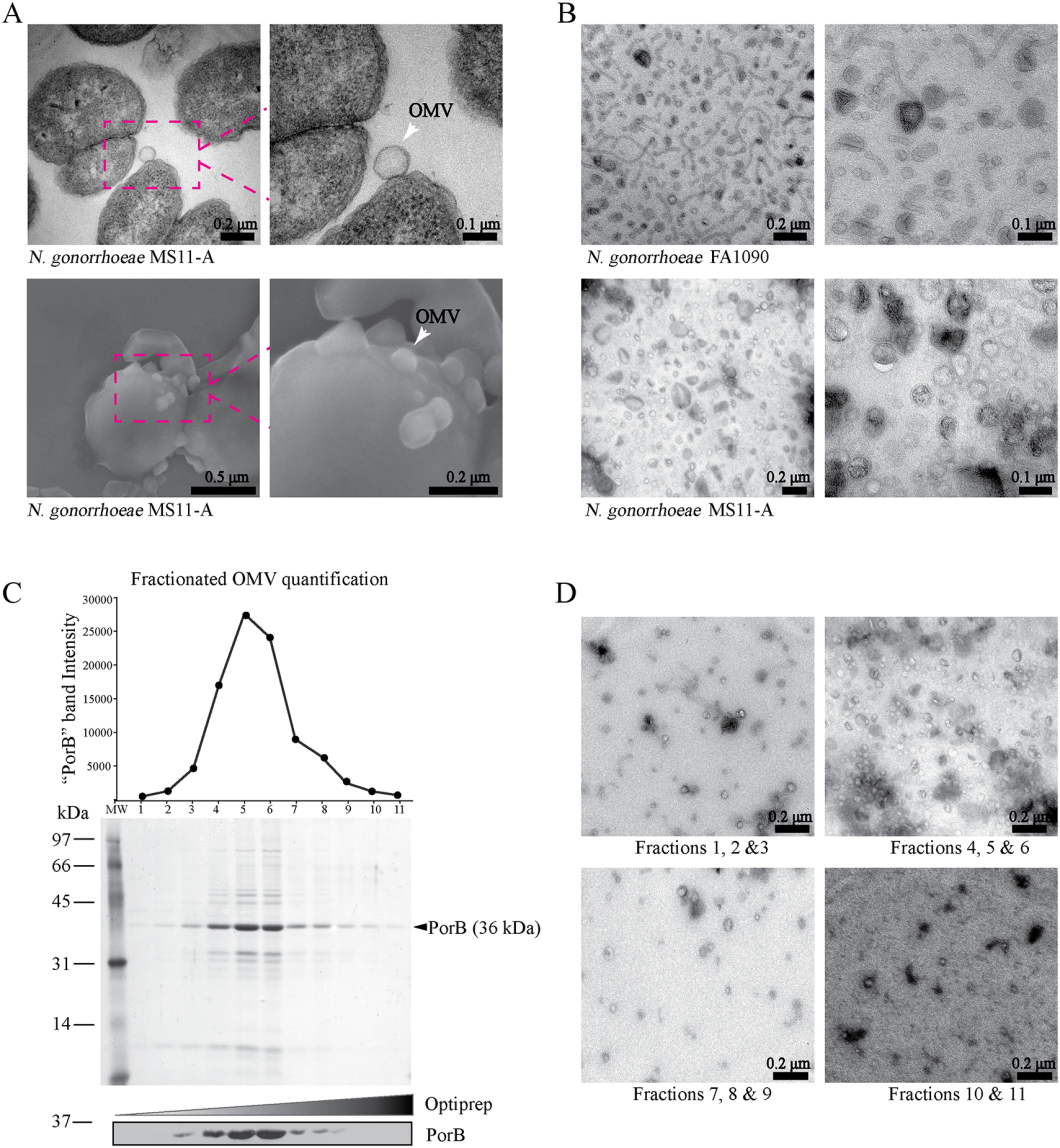
Purification of *N*. *gonorrhoeae* secreted OMVs. (A) *N*. *gonorrhoeae* MS11-A was analyzed by transmission (top panels) and scanning (bottom panels) electron microscopy (EM). Arrows indicate OMVs. Scale Bar = 0.5, 0.2 and 0.1μm. (B) Crude OMV preparations from FA1090 and MS11-A cultures were analyzed by transmission electron microscopy (TEM) (right panels show higher magnification images) (C) *N*. *gonorrhoeae* MS11-A derived OMVs were fractionated by OptiPrep density gradient ultracentrifugation and 11 fractions and proteins analyzed by colloidal coomassie staining. Densitometry of the prominent band that corresponds to PorB (shown on the top). Immunoblot analysis of each of the fractions probed with anti-PorB (bottom). Molecular weight markers (kDa) are indicated on the left. (D) TEM images of the negatively stained pooled fractions from OptiPrep density gradient as indicated at the bottom. Scale Bar = 0.2 μm.

To study the role of *N*. *gonorrhoeae* secreted OMVs, we first established methods for the purification of OMVs under optimal growth conditions to minimize bacterial autolysis and contamination with membranous and cytosolic materials, all of which are affected by culture conditions [30]. Transmission electron microscopy (TEM) and scanning electron microscopy (SEM) of *N*. *gonorrhoeae* harvested from mid-log phase cultures demonstrated that the bacterial membrane remained largely intact (Fig 1A). TEM and SEM also detected vesicles that appeared closely associated with the outer membrane of *N*. *gonorrhoeae* and emerging from the bacterial surface (Fig 1A). OMVs collected from *N*. *gonorrhoeae* growth media by centrifugation contained intact membranes and ranged in size (20–200 nm diameter), shape (from spherical to tubular) and in the number of membranes (mono or bi-layers) from two different strains (Fig 1B). The pelleted OMVs also contained electron dense particles, which may reflect protein aggregates (Fig 1B). To further purify OMVs with defined composition and uniform size from culture supernatants, we employed 25–50% OptiPrep gradients. After centrifugation, fractions 4, 5 and 6 contained several proteins whereby the dominant protein was likely the outer membrane porin, PorB, based on the molecular weight and immune detection with anti-PorB serum (Fig 1C). TEM analysis of fractions 4, 5 & 6 contained OMVs with uniform size of 50–100 nm in diameter and a single bilayer in the absence of cellular debris (Fig 1D). In contrast, fractions 1–3 and 7–10 contained electron-dense structures as well as membrane vesicles with varying shapes and sizes (Fig 1D). For subsequent analysis, fractions 4, 5 & 6 obtained from log-phase *N*. *gonorrhoeae* cultures after OptiPrep ultracentrifugation were used as highly enriched populations of intact OMVs, whereas total vesicles from culture supernatants were designated as crude OMV preparations.

### Proteins are selectively enriched or omitted in *N*. *gonorrhoeae* secreted OMVs

**Fig 2:**
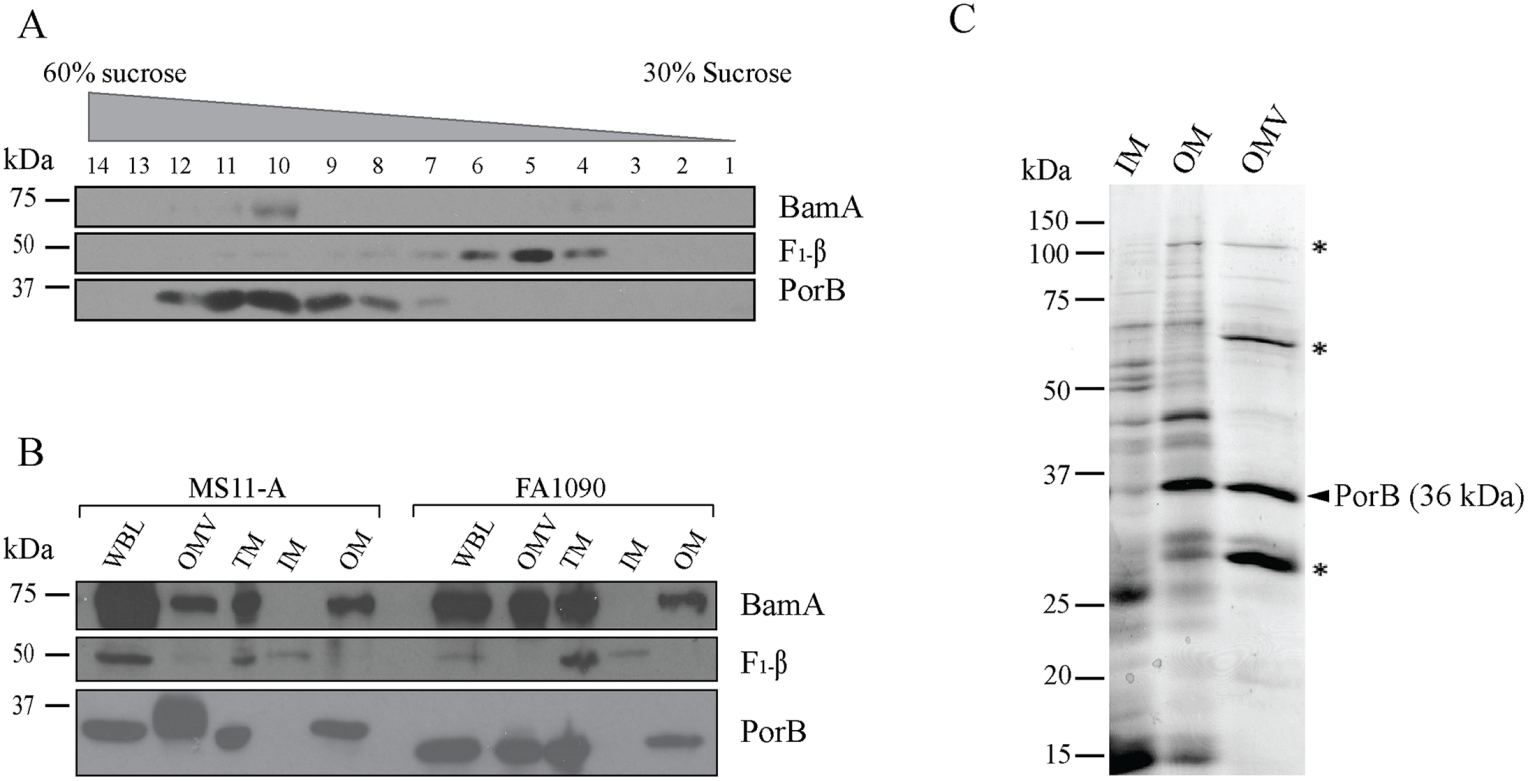
OMVs secreted by *N*. *gonorrhoeae* contain distinct proteins. (A) Total membranes of *N*. *gonorrhoeae* were separated by sucrose gradient ultracentrifugation and fractions were probed with anti-PorB and anti-BamA to detect fractions with outer membranes and anti-F_1_-β with inner membranes. Fractions 10 and 5 were used as outer and inner membrane fractions, respectively, in further analysis. (B) OMVs, outer membrane (OM), inner membrane (IM), total membrane (TM) and whole bacterial lysate (WBL) were probed for two outer membrane proteins (BamA and PorB) and inner membrane protein, F_1_-β. (C) Colloidal coomassie staining of proteins of *N*. *gonorrhoeae* secreted outer membrane vesicles (OMV), outer membrane (OM) and inner membrane (IM). The positions of molecular size markers (kDa) are shown on the left. PorB (36 kDa) is indicated on the right and over represented proteins in OMVs by asterisks.

OMVs originate from bacterial outer membranes [23]. To determine whether the protein content of purified OMVs is thus shared with outer membranes in *N*. *gonorrhoeae*, inner and outer membranes were separated using discontinuous sucrose gradient ultracentrifugation. Fraction 10 and 5 were enriched for outer and inner membranes, respectively, as they contained outer membrane proteins PorB and BamA (fraction 10) and the inner membrane protein F_1_β (fraction 5) (Fig 2A). Immunoblot analysis confirmed that the outer membrane proteins, BamA and PorB, are present in purified OMV and outer membrane fractions, whereas F_1_β was detectable on inner membrane fractions but absent from the outer membrane fractions of two different *N*. *gonorrhoeae* strains (Fig 2B). This confirms that outer membrane proteins are major components of OMVs. However, the relative proportions of proteins identified with colloidal coomassie staining indicated differences in the specific protein composition of outer membrane and OMVs (Fig 2C). This includes both over-representation and selective absence of different proteins. Overall, this demonstrates that OMVs secreted by *N*. *gonorrhoeae* share proteins with the outer membrane, but the relative protein composition of OMVs differs from the outer membrane.

### The proteome of purified *N*. *gonorrhoeae* secreted OMVs

**Fig 3:**
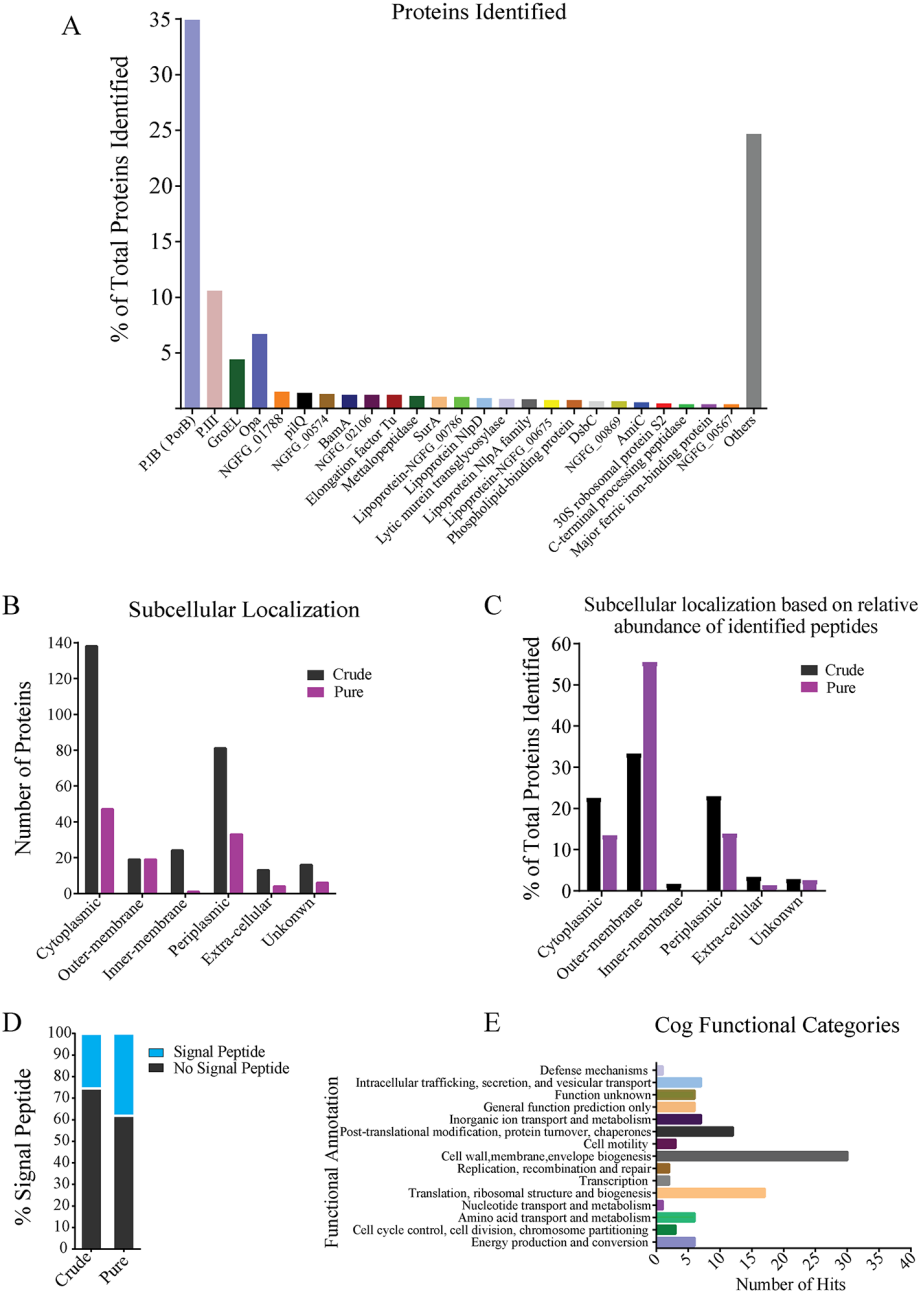
Porins are the major membrane proteins identified in purified OMVs. (A) Proteome of purified *N*. *gonorrhoeae* MS11-A OMVs as identified by LC-MS/MS relative abundance based on peptide matched spectra. (B) Predicted subcellular localization of the number of identified proteins in purified and crude OMVs. (C) Predicted subcellular localization of identified proteins in purified and crude OMVs based on the relative abundance of identified peptides. (D) The relative percentage of proteins containing signal peptides in purified and crude OMVs, based on SignalP v.4.1 analysis. (E) All identified proteins in purified OMVs were functionally classified based on COG database using WebMGA server.

To gain insights into the function of *N*. *gonorrhoeae* OMVs, we determined the protein content in more detail by mass spectrometry. This identified 110 proteins with high confidence in purified OMVs, compared to 291 in crude OMV fractions from culture supernatants (S1 Table). Based on the percentage share of mass spectra of identified peptides, the 25 most abundant proteins (constituting 75% of total peptide matched spectra) identified in the purified OMV preparation are listed and represented in Fig 3A. PorB peptide matched MS/MS spectra added up to 35% of the peptide matched spectra of all proteins identified on purified OMV preparation and PorB was the most dominant protein based on spectral counting, followed by P.III and Opa (opacity) proteins. These well-known group of *N*. *gonorrhoeae* outer membrane proteins comprised the majority (~60%) of all OMV derived peptide matched spectra (Fig 3A). OMVs contained additional outer membrane proteins including adhesion protein MafA, the β-barrel assembly machinery BamA, cell division proteins FtsN and AmiC, but also several periplasmic proteins (e.g. SurA, NlpD, NlpA and metallopeptidase). A few highly abundant cytoplasmic proteins, such as GroEL, 30S ribosomal protein S2 and elongation factor Tu were present (Fig 3A, S1 Table). In addition, we detected 10 uncharacterized proteins on purified OMVs (S1 Table). In contrast, the crude OMV fraction contained additional proteins associated with pili and fimbriae formation, translation and transcription machinery, transport, as well as iron-binding proteins and cytosolic catalase (S1 Table).

Comparative analysis of protein subcellular localization revealed that almost 100 cytoplasmic proteins are excluded after OMV purification (Fig 3B and S2 Table). Also, only one putative inner membrane protein (D1DJS8, annotated as “sulfatase”) was identified on purified OMVs compared to 24 inner membrane proteins on crude fractions (Fig 3B and S1 Table). Similarly, 6 and 16 proteins of unknown subcellular localization were identified on purified and crude OMV preparation, respectively (Fig 3B). Furthermore, annotation for subcellular localization of identified protein on purified and crude OMVs based on the relative abundance of identified peptides revealed that the majority of the proteins on purified OMV preparations are derived from outer-membranes, comprising 55% of the total OMV proteome. Notably, outer-membrane proteins constituted only 33% of the total proteins identified on crude OMV preparation (Fig 3C). Purification of OMVs also increased the relative abundance of protein containing canonical secretions signals (38% compared to 28% in crude OMV preparations) (Fig 3D). Finally, the majority of identified proteins on purified OMVs were predicted to be involved in cell wall, membrane and envelope biogenesis (Fig 3E). Taken together, our data demonstrates that highly purified OMVs from *N*. *gonorrhoeae* contain abundant levels of outer membrane proteins, including PorB, and show reduced levels of cytoplasmic and inner membrane proteins, commonly associated with OMVs obtained from crude culture supernatants.

### PorB is associated with the membrane of *N*. *gonorrhoeae* OMVs

**Fig 4:**
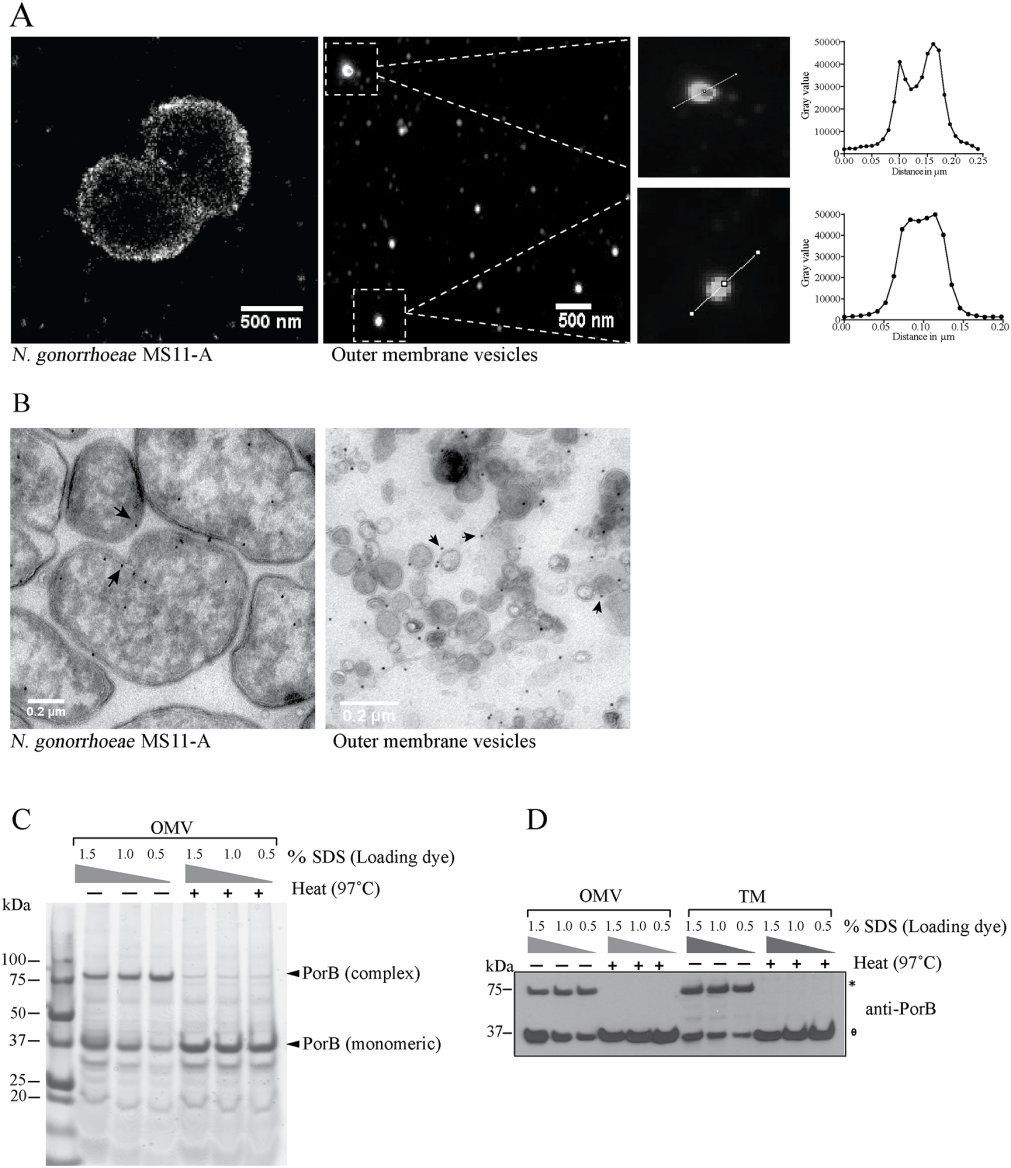
PorB complex formation in OMVs. (A) Super-resolution microscopy (dSTORM) of *N*. *gonorrhoeae* (left) and outer membrane vesicles (middle and zoomed in right) probed with anti-PorB serum. Panel on the right shows fluorescence intensity along the line across vesicles. Scale bar = 500 nm. (B) Transmission electron microscopy of *N*. *gonorrhoeae* (left panel) and OMVs (right panel) probed for PorB using 10 nm-gold conjugated antibodies. Gold particle distribution around the surface of bacteria and vesicles represented by arrow. Scale bar = 0.2 μm. (C) Boiled and unboiled purified OMVs were solubilized with increasing concentrations of detergent (sodium dodecyl sulfate, SDS) as indicated and analyzed by colloidal coomassie staining after semi-native gel electrophoresis. Monomeric and a higher order molecular weight PorB protein complex are indicated by arrows. (D) Immunoblot analysis of OMVs and bacterial total membranes isolated from *N*. *gonorrhoeae* with increasing concentrations of detergent (SDS) as indicated and probed for PorB after semi-native gel electrophoresis. Monomeric (ɵ) and higher order molecular weight PorB protein complex (*) are indicated.

Having established that PorB was the major protein found on purified OMVs, we next investigated whether it was folded and associated with the OMV membrane or packaged as unfolded amino acid chain within the vesicles. For this, PorB localization on whole bacterial cells and purified OMVs was determined by super-resolution microscopy (direct-stochastic optical reconstruction microscopy—dSTORM). As expected, single molecule signals of PorB were concentrated around the periphery of whole bacterial cells, consistent with outer membrane localization (Fig 4A). Similarly, PorB was detected on OMVs whereby bigger sized vesicles (around 100 nm diameter) showed ring like structures (Fig 4A). Analysis of fluorescent signal intensity across PorB labelled OMVs were consistent with membrane localization (Fig 4A). The majority of OMVs, however, did not show distinct membrane staining, most likely due to the small size and limited resolution (Fig 4A). Thus, the localization of PorB was determined by TEM after immunogold labelling with anti-PorB serum. Electron microscopy of ultrathin sections were consistent with outer membrane localization of PorB in *N*. *gonorrhoeae* (Fig 4B). Similarly, gold particles associated primarily with membranes rather than with the luminal content of OMVs (Fig 4B).

Next, we determined whether PorB existed as a protein complex in OMVs, as was recently shown in the outer membrane of *N*. *gonorrhoeae* [15]. A protein complex of PorB (~75 kDa) was readily detectable in OMVs and total membrane fractions by semi-native polyacrylamide gel electrophoresis after coomassie staining or immunoblot analysis (Fig 4C and 4D). As expected, increasing concentration of SDS or boiling prior to analysis dissociated the PorB complex isolated from OMVs and total membrane fractions into its monomeric form (36 kDa) (Fig 4C and 4D). This demonstrates, that PorB adopts a similar native conformation in OMVs as observed in the outer membrane of *N*. *gonorrhoeae*.

### Secreted OMVs enable PorB targeting of mitochondria in macrophages

**Fig 5:**
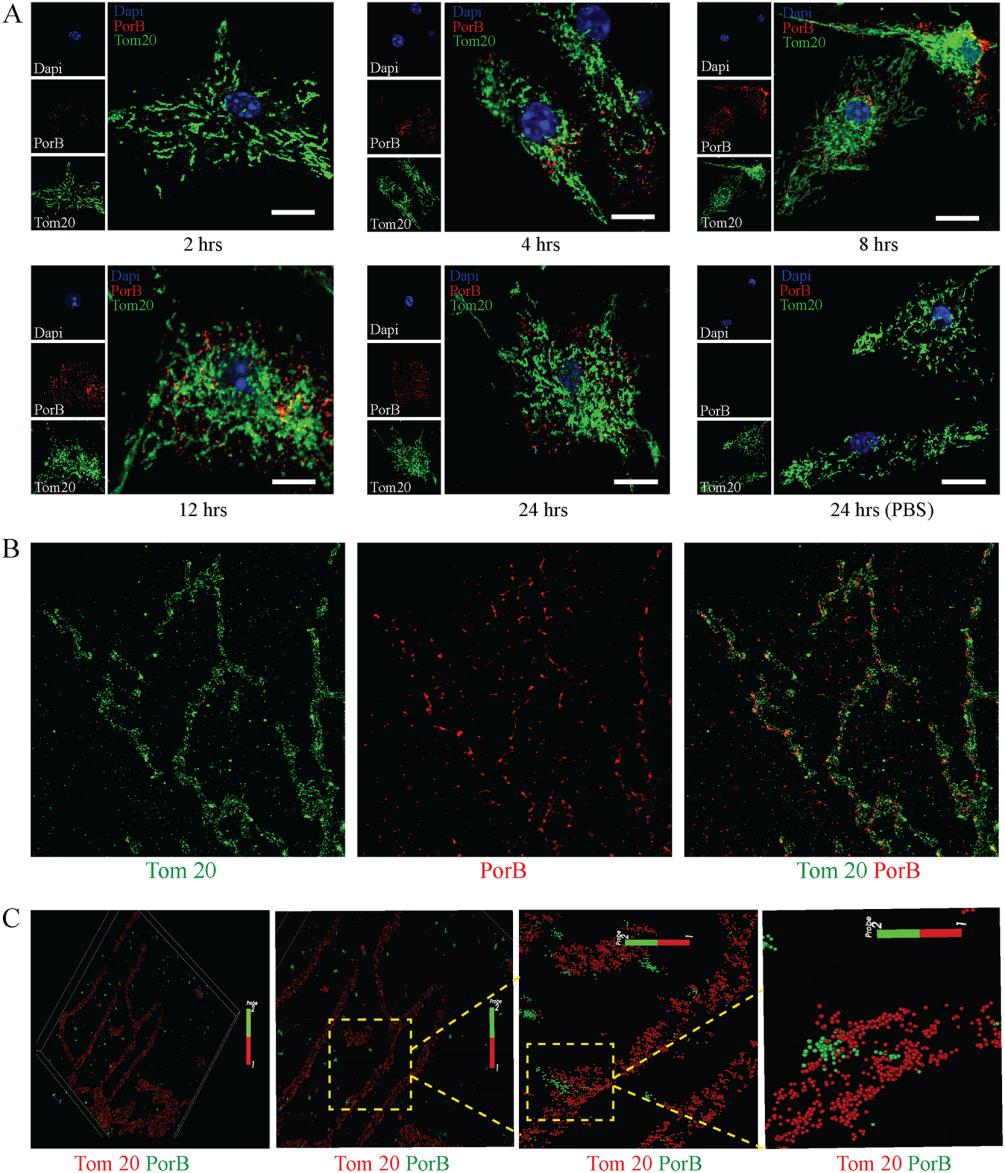
OMVs enable mitochondria targeting of PorB. (A) Bone marrow-derived macrophages (BMDMs) were treated with purified *N*. *gonorrhoeae* OMVs and analyzed by confocal laser scanning microscopy for PorB (red) and Tom20 (green) localization at indicated times. Cells were stained with DAPI (blue) to visualize nuclei. Scale bar = 10 μm. Representative images of more than 200 cells from three biological samples. (B) BMDMs treated with purified OMVs for 12 hours were analyzed by RapidSTORM reconstructed dual colour super-resolution imaging for PorB (red) and Tom20 (green) localization. (C) OMV treated BMDMs (12 hours) were probed with anti-PorB (green) and Tom20 (red) antibodies and analyzed by 3D single-molecule localization super-resolution microscopy.

**Fig 6:**
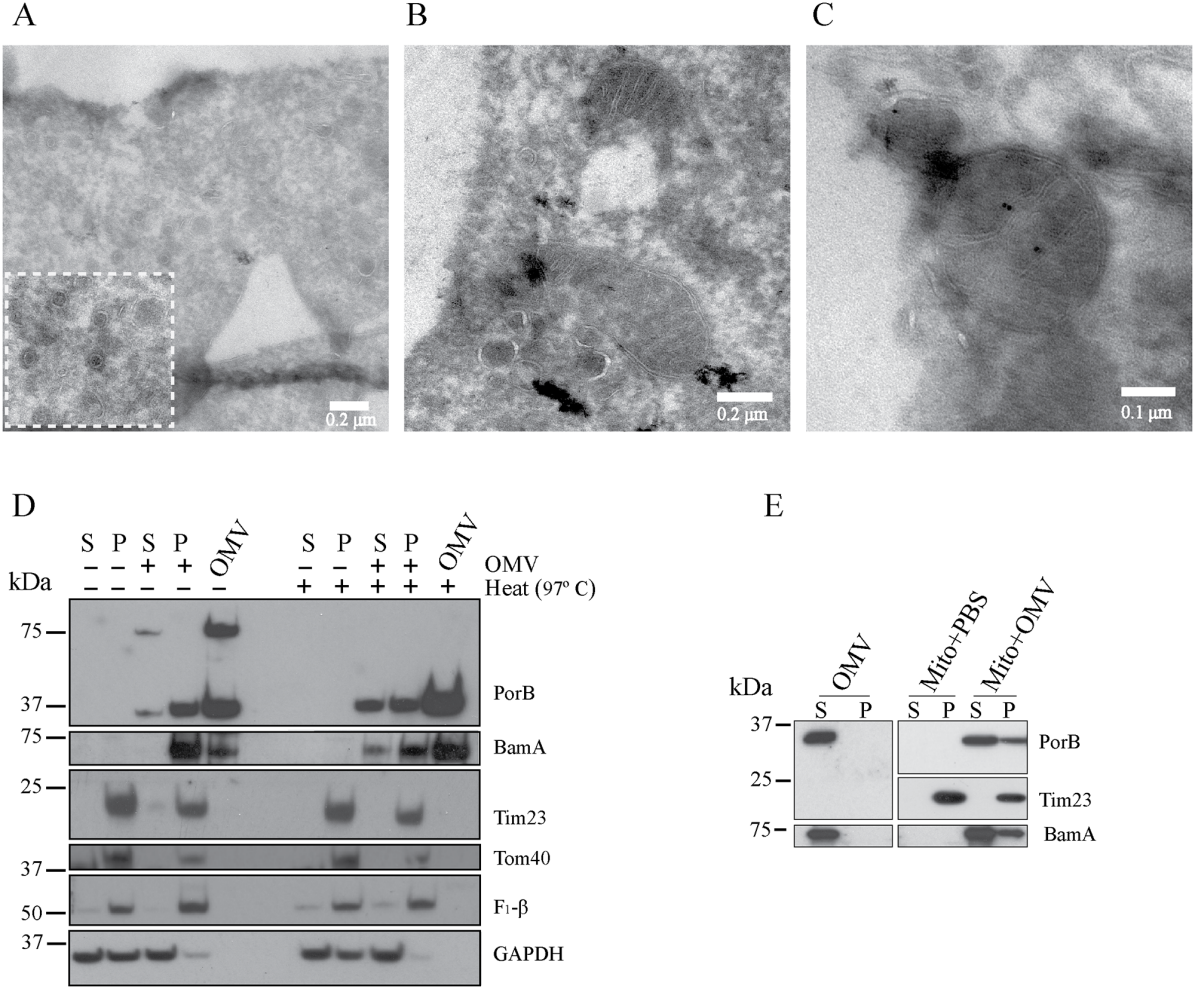
OMVs associate with mitochondria. (A, B and C) Bone marrow-derived macrophages (BMDMs) were treated with purified *N*. *gonorrhoeae* OMVs for 24 hours and imaged by transmission electron microscopy by negative staining and (C) 10 nm-gold conjugated antibodies against PorB. (D) Crude mitochondria (P) and cytoplasmic fractions (S) were obtained from OMV-treated BMDMs and analyzed for PorB and BamA and mitochondria (Tim23, Tom40 and F_1_β) and cytosolic (GAPDH) markers by immunoblot analysis after semi-native gel electrophoresis. (E) Mitochondria-enriched fractions were incubated with purified OMVs or PBS and subsequent supernatant (S) and pellet (P) fractions were analyzed by immunblotting with anti-PorB, BamA and Tim23 antibodies after SDS gel electrophoreses.

Given that PorB was an abundant protein in OMVs and that ectopic expression of PorB in mammalian cells resulted in mitochondria localization, we determined whether *N*. *gonorrhoeae* vesicles enabled mitochondria trafficking of the porin. For this, we exposed bone-marrow derived macrophages (BMDMs) with *N*. *gonorrhoeae* purified OMVs and determined co-localization of PorB with Tom20, an outer mitochondrial membrane protein, by confocal microscopy. At early time points (2, 4 and 8 hours), PorB mainly localized to perinuclear regions, with little evidence of co-localization with Tom20 (Fig 5A). While by 12 and 24 hours post-treatment PorB remained in a punctated pattern, it was dispersed throughout the cell (Fig 5A). At these time points, the PorB signal appeared in close proximity to mitochondria, but resulted in only partial co-localization with Tom20 by confocal microscopy (Fig 5A). Similar results were obtained with human THP-1 macrophages, whereby PorB was detected in a punctated pattern in close proximity to mitochondria after OMV exposure (S1 Fig). To increase the detection of low levels of PorB that might have targeted mitochondria, the mitochondrial network was further visualized by super-resolution microscopy, using dSTORM. In OMV treated BMDMs, PorB localized as discrete patches to the Tom20 stained mitochondrial network at 12 hours post treatment (Fig 5B). Using fast 3D video-rate, super-resolution single-molecule localization resolved the outer mitochondrial membranes based on Tom20 staining (Fig 5C). Furthermore, PorB molecules were embedded within a Tom20 stained membrane (Fig 5C). Again, the PorB signal remained in distinct clusters rather than dispersed throughout the mitochondrial membrane. This suggested that OMVs may target PorB directly to mitochondria. In agreement with this, we observed OMVs within the cytosol of macrophages by electron microscopy at 24 hours post treatment, as OMVs lacked surrounding host membranes (Fig 6A). Furthermore, OMVs were detectable at close proximity to mitochondria (Fig 6B), and the membranes of OMVs and mitochondria were stained positive for PorB (Fig 6C). Crude fractionation of mitochondria from the remaining cytoplasm further indicated that PorB associated with mitochondria (Fig 6D). As observed by electron microscopy, a proportion of PorB was associated with the remaining cytoplasmic fraction (Fig 6D). Intriguingly, mitochondria targeted PorB was exclusively monomeric as evidenced by semi-native polyacrylamide gel electrophoresis (Fig 6D). PorB in the remaining cytoplasmic fraction was detectable as an oligomeric complex which was sensitive to heat treatment (Fig 6D). We thus hypothesized that OMVs may directly transfer PorB to mitochondria and potentially other OMV cargo proteins. Indeed, we also detected BamA within the mitochondria-enriched fraction of OMV-treated BMDMs (Fig 6D). To mimic OMV targeting of mitochondria, we isolated mitochondria from untreated BMDMs and incubated them with purified OMVs *in vitro*. As observed in OMV-treated cells, a detectable proportion of PorB and BamA fractionated with isolated mitochondria *in vitro*, but failed to pellet in the absence of mitochondria (Fig 6E). This suggests that *N*. *gonorrhoeae* OMVs are targeted to the cytosol of macrophages and closely associate with mitochondria to deliver PorB and other OMV proteins.

### PorB targeting to macrophages depends on OMV biogenesis

**Fig 7:**
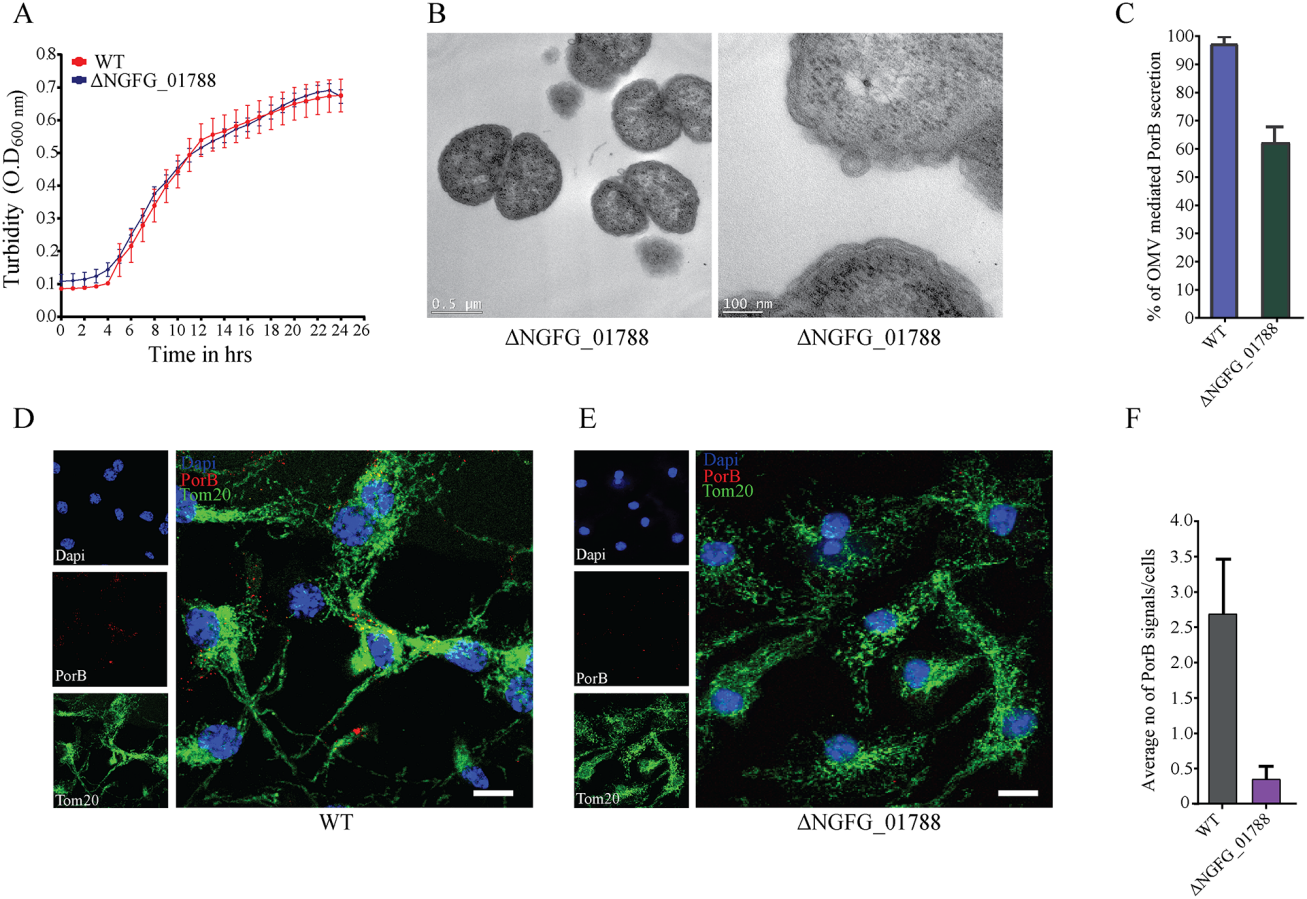
*N*. *gonorrhoeae* depends on OMVs for PorB delivery to macrophages. (A) Wild type (WT) and ΔNGFG_01788 deletion mutant were cultured in GC media and growth was monitored by optical density at 600 nm. Mean and standard deviation from triplicate experiments. (B) Transmission electron microscopy of ΔNGFG_01788 mutant showing diplococci and OMV secretion. (C) Purified OMVs and whole bacteria lysates were analyzed by coomassie staining after SDS gel electrophorese and the amount of PorB within OMVs relative to whole bacterial cells determined by densitometry. Relative OMV-mediated secretion of PorB in the mutant is based on wild type levels (100%). Mean and standard deviation from three independent experiments are shown. (D) BMDMs were incubated with wild type *N*. *gonorrhoeae* or (E) ΔNGFG_01788 mutant in transwells and analyzed for PorB (red) and Tom20 (green) localization by immunofluorescence analysis after 24 hours. DAPI (blue) indicates cell nucleus. (F) The number of PorB signals (puncta) was quantified from >100 cells and two independent experiments.

The biogenesis of OMVs remains poorly characterized, but several studies have now identified bacterial factors that impact on the rate of vesicle secretion [23]. In part, OMV biogenesis depends on the interaction of the outer membrane with the underlying peptidoglycan. Thus, we reasoned that the gene-product of *N*. *gonorrhoeae* NGFG_01788 may affect OMV biogenesis as it contains a carbohydrate-binding lysin motif (LysM) and was readily detectable on purified OMVs (Fig 3A). Genetic deletion of NGFG_01788 did not affect *N*. *gonorrhoeae* growth in rich culture media or the formation of diplococci (Fig 7A and 7B). NGFG_01788 mutants also produced OMVs, but OMV-mediated PorB secretion was reduced by 40% compared to the parental wild type strain, as determined by coomassie stained PorB from OMV and whole cell lysate fractions (Fig 7C and S2A Fig), consistent with an overall reduced rate of OMV biogenesis (S2B Fig). Next, we incubated wild type and mutant bacteria in transwells that enable physical separation from BMDMs, but allow small vesicles to move freely. After 24 hours, we harvested macrophages and determined PorB localization by confocal microscopy. As observed with purified OMVs, we were able to detect PorB in a punctated pattern in close proximity to mitochondria in BMDMs (Fig 7D). Compared to wild type *N*. *gonorrhoeae*, PorB was detected at reduced rates in BMDMs after incubation with the NGFG_01788 mutant (Fig 7F). This demonstrates that *N*. *gonorrhoeae* is able to deliver PorB to host cells in the absence of bacterial attachment and suggests that this depends on OMV biogenesis.

### *N*. *gonorrhoeae* OMVs induce macrophage apoptosis

**Fig 8:**
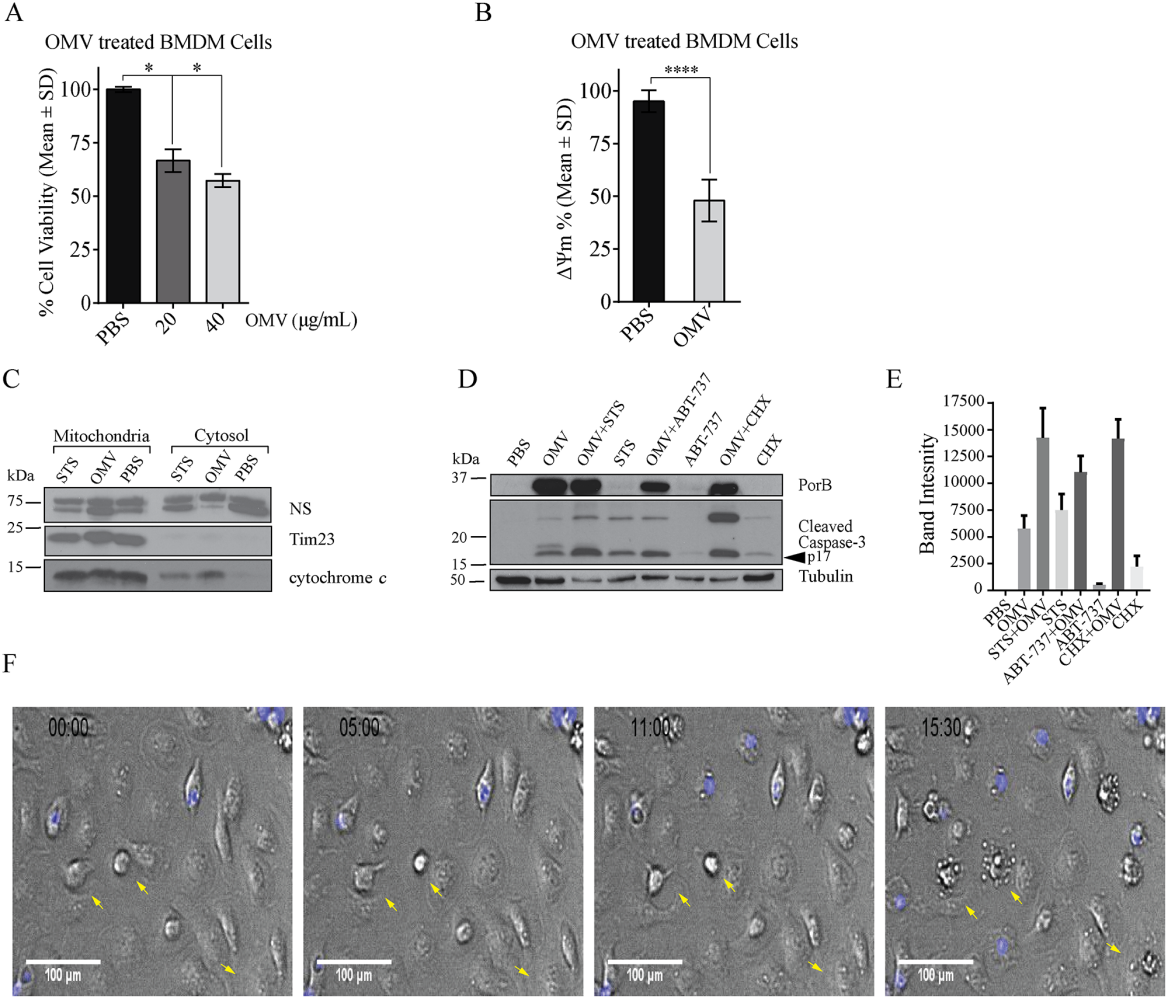
OMVs trigger loss of mitochondrial health and activation of apoptotic caspases in macrophages. (A) BMDMs treated with *N*. *gonorrhoeae* OMVs (20 and 40 μg/mL) for 48 hours were analyzed by the MTT assay to determine cell viability and mitochondrial activity relative to PBS treated cells. Mean and SD from 3 independent experiments are shown (asterisks indicate p<0.01). (B) BMDMs were labelled with TMRM and treated with purified OMVs (40 μg/mL) for 48 hours to determine the mitochondrial membrane potential (ΔΨm) compared to PBS treated cells. Mean and SD from three independent experiments, containing triplicate biological samples with more than 500 cells (asterisks indicate p<0.001). (C) BMDMs were treated with staurosporine (STS), OMVs and PBS for 24 hours and mitochondria and cytosolic fraction were analyzed by immunoblotting for cytochrome *c* and Tim23. A nonspecific (NS) band from anti cytochrome *c* antibody was used as a control for loading. (D) BMDMs were treated with PBS, OMVs, STS, ABT-737 and cycloheximide (CHX) for 24 hours and analyzed by immunoblotting for cleaved caspase-3 (p17 kDa). Anti-PorB and anti-tubulin were used as control for OMV treated cells and loading, respectively. Data representative of three independent experiments. (E) Quantification of cleaved caspase-3 (p17 kDa) from panel D by densitometry. Mean and SD from three independent experiments shown. (F) OMV treated BMDMs were incubated with Draq7 (blue) to stain nuclei of dead cells and analyzed by time lapse imaging. Time frames are shown. Arrows indicate blebbing cells. Scale bar = 100 μm.

Given that OMV-derived PorB targeted mitochondria, we next determined mitochondrial health of OMV exposed BMDMs. For this, BMDMs were treated with different concentrations of OMVs and mitochondrial activity measured by reduction of T (3[4, 5 dimethylthiazol-2-yl]-2, 5-diphenyl tetrazolium bromide (MTT). Compared to vehicle control (PBS) treated BMDMs, cells exposed to 20 and 40 μg/mL purified OMVs showed significant decreased metabolic activity (66.62% +/- 5.34 and 57.29%+/-3.04, respectively; p<0.05) (Fig 8A). Mitochondria membrane potential (ΔΨm), as determined by TMRM staining, was reduced by 50% in OMV treated BMDMs (Fig 8B). Translocation of PorB to mitochondria and dissipation of ΔΨm lead us to further investigate if OMV exposure resulted in cytochrome *c* release and activation of intrinsic apoptosis. As expected, cytochrome *c* was found in the mitochondria containing fractions, and not the cytosol, in untreated BMDMs (Fig 8C). In contrast, staurosporine (STS) treatment, which induces apoptosis, caused cytochrome *c* release into the cytosol (Fig 8C). Similarly, cytochrome *c* was markedly associated with cytosolic fraction in OMV-treated BMDMs, while the inner mitochondrial membrane protein, TIM23, remained associated with mitochondrial fractions (Fig 8C).

Cytosolic cytochrome *c* causes the proteolytic cleavage and activation of the cell death executioner caspases, caspase-3 and 7. Cleaved caspase-3 was only detectable in BMDMs after STS and cycloheximide (CHX) treatment, known inducers of apoptosis, but not in PBS control treated cells (Fig 8D). Similarly, OMV exposure triggered cleavage of caspase-3 in BMDMs, which contained PorB (Fig 8D). Consistent with caspase-3 activation, membrane blebbing, which is characteristic of apoptotic death, was detectable at around 15 hours after incubation of BMDMs with OMVs (Fig 8F). Co-treatment of BMDMs with OMVs and STS further increased the levels of cleaved caspase-3, indicating that they act synergistically to induce apoptosis (Fig 8D and 8E). Similarly, OMVs triggered increased levels of cleaved caspase-3 in the presence of CHX, suggesting that synthesis of at least one host protein can limit apoptosis signaling under these conditions (Fig 8D and 8E). To test whether this was dependent on pro-survival BCL-2 family members that prevent mitochondria mediated apoptosis, BMDMs were treated with ABT-737 to inhibit BCL-2, BCL-XL and BCL-W proteins. As shown recently [31], ABT-737 by itself did not lead to caspase-3 cleavage due to the presence of pro-survival BCL-2 related proteins that are resistant to the compound. However, ABT-737 did lead to increased caspase-3 cleavage in OMV treated macrophages (Fig 8D and 8E), suggesting that *N*. *gonorrhoeae* OMVs are taken up by macrophages and induce mitochondrial damage and BCL-2 mediated apoptosis.

### PorB containing OMVs cause mitochondrial damage and apoptosis over time

**Fig 9:**
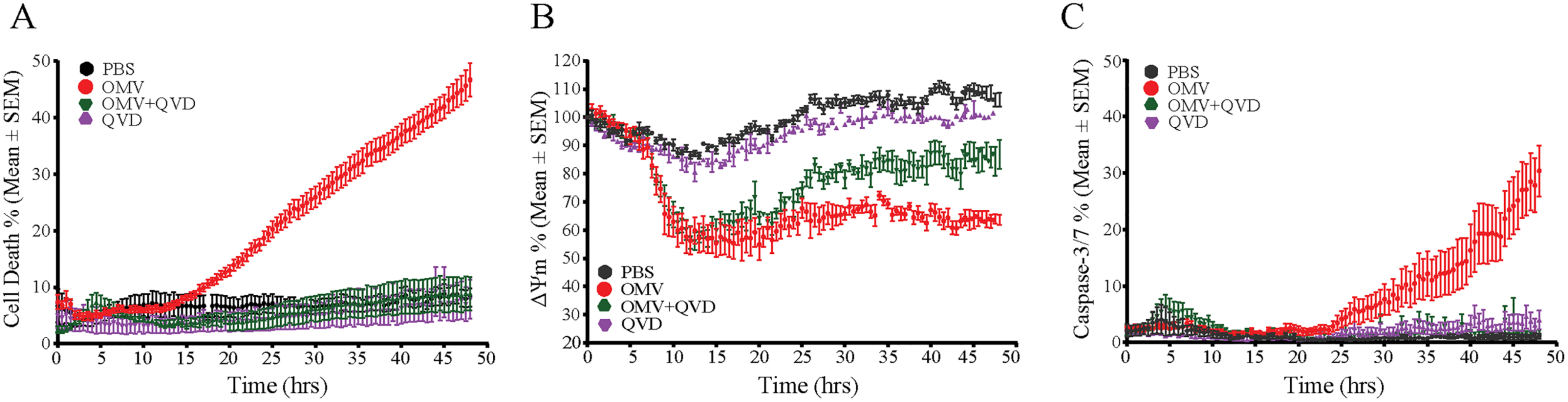
OMVs induce delayed macrophage death. BMDMs treated with PBS, *N*. *gonorrhoeae* purified OMVs and the pan-caspase inhibitor, Q-VD-PH (QVD) were analyzed by live-cell imaging every 30 minutes for 48 hours to determine (A) cell death (Draq7 positive cells), (B) mitochondrial membrane potential (ΔΨm, TMRM positive cells) and (C) caspase-3/7 activity (caspase fluorogenic substrate positive cells). Mean and standard error of the mean (SEM) are shown for three independent experiments, containing triplicate biological samples.

To follow macrophage and mitochondrial health on the single cell level over time, we established time-lapse imaging of OMV treated BMDMs that enabled visualization of cellular morphology, mitochondrial membrane potential (ΔΨm) via TMRM fluorescence, caspase-3/7 activity using fluorescent probes and macrophage viability via staining with the membrane impermeable DNA dye, Draq7, in real time. PBS treated BMDMs remained viable (> 90% Draq7 negative and TMRM positive) over 48 hours of imaging every 30 minutes (Fig 9A and 9B). In contrast, 40% of OMV-treated BMDMs lost the TMRM signal within 10 hours (Fig 9B and S1 and S2 Videos). OMV exposure also caused plasma membrane rupture and macrophage death overtime starting 15 hours post treatment (Fig 9A). Consistent with apoptotic death, the majority of dead macrophages showed caspase-3/7 activity (Fig 9C and S1 Video). Of note, the caspase-3/7 activity is transient (1–2 hours), in contrast to Draq7 staining (stable for 48 hours), and thus indicates the number of cells with active caspases at each time frame rather than over extended periods of time. Single cell analysis confirmed that macrophage death is preceded by loss of TMRM signal and caspase-3/7 activity (S3 Fig). In addition, the pan-caspase inhibitor Q-VD-PH (QVD), which inhibits apoptotic caspase activity, prevented OMV induced cell death and caspase-3/7 activity, but not loss of ΔΨm, in BMDMs (Fig 9A, 9B and 9C).

### PorB is sufficient to trigger apoptosis in macrophages

Given the OMVs contain over 100 proteins, we wanted to confirm that PorB is the major bacterial factor that causes apoptosis. We were unable to inducibly reduce or deplete PorB in *N*. *gonorrhoeae*, consistent with the notion that the gene is essential [16]. Alternatively, we ectopically expressed native PorB in HeLa cells under a doxycycline inducible promoter (as similar approaches remained unsuccessful in macrophages). PorB co-localized with the mitochondrial marker, Tom20, within 2 hours after doxycycline induction and caused mitochondrial fragmentation by 8 hours in cells containing detectable PorB (S4A Fig). PorB expression triggered caspase-3 cleavage, as observed after OMV treatment (S4B Fig). Caspase-3 cleavage was not detected in the absence of doxycycline or PorB containing plasmids (S4B Fig). We also determined whether PorB was detectable as monomeric protein or whether it formed an oligomeric complex. We only detected ectopically expressed native PorB as a monomeric protein (S4C Fig), similar to mitochondria-associated PorB after OMV treatment. This demonstrates that PorB is sufficient to induce apoptosis and suggests that PorB adopts a monomeric, rather than oligomeric, conformation in mitochondria.

**Fig 10:**
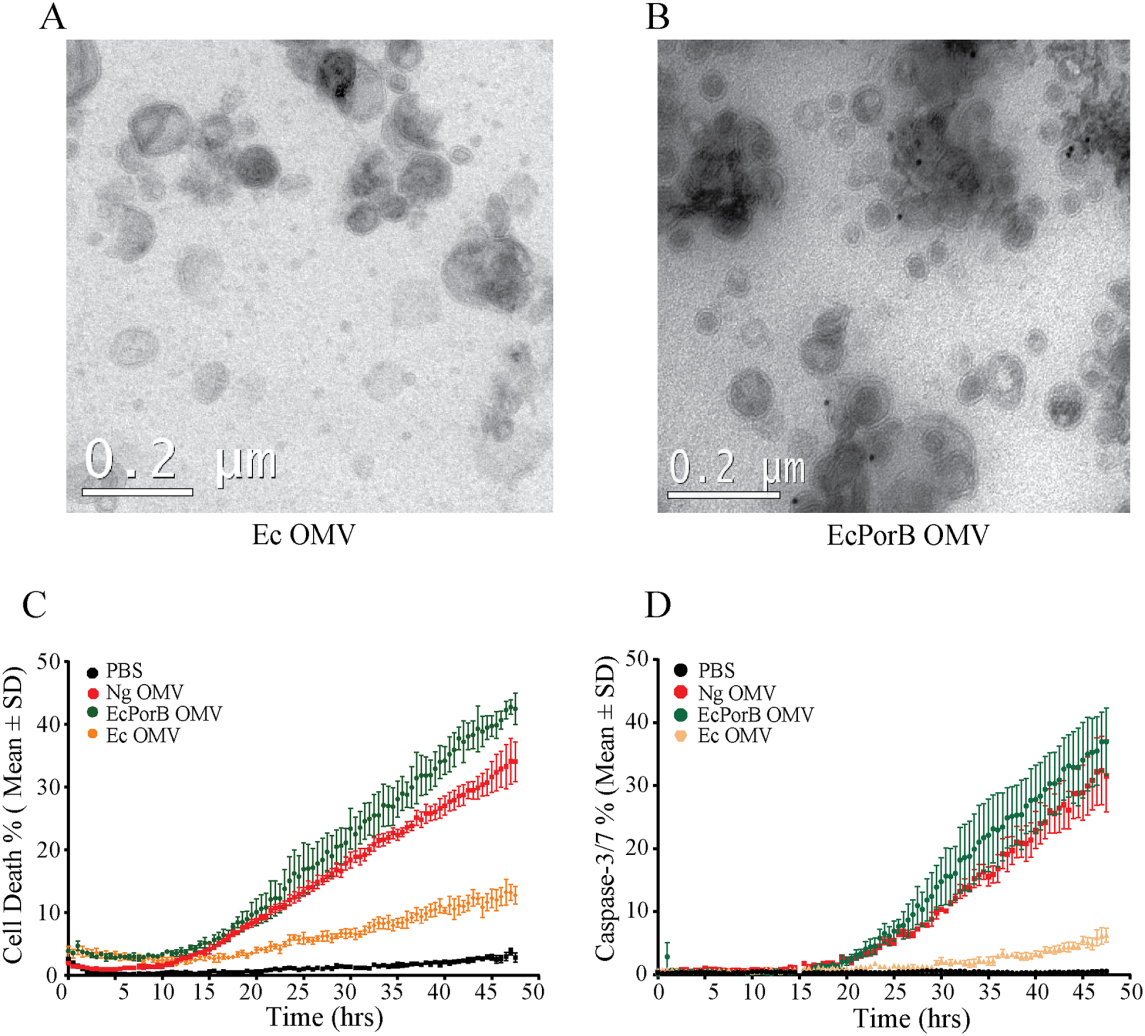
PorB delivered by *E*. *coli* OMVs induces macrophage apoptosis. (A and B) *N*. *gonorrhoeae* PorB was expressed in *E*. *coli* and OMVs from *E*. *coli* (Ec OMV) and PorB expressing *E*. *coli* (Ec PorB OMV) analyzed by transmission electron microscopy and anti-PorB immunogold labelling. (C and D) BMDMs were treated with PBS and OMVs from *N*. *gonorrhoeae* (Ng OMV), PorB expressing *E*. *coli* (Ec PorB OMV) or control *E*. *coli* (Ec OMV) and (C) cell death (Draq7) and (D) caspase-3/7 activity (fluorogenic substrate) was determined every 30 minutes for 48 hours using live-cell imaging. Mean and standard deviation from two independent experiments with three biological repeats are shown.

To further verify that OMV-delivered PorB is able to cause apoptosis, we expressed *N*. *gonorrhoeae* PorB in non-pathogenic *Escherichia coli*. Electron microscopy indicated that OMVs from PorB expressing *E*. *coli* (*Ec* PorB OMV) contained membrane-associated porins, as observed with *N*. *gonorrhoeae* purified OMVs (Fig 10A and 10B). Treatment of BMDMs with *Ec* PorB OMV caused macrophage death after a delay of 15 hours to a similar extend as *N*. *gonorrhoeae* OMVs (Fig 10C). *Ec* PorB OMV also caused the activation of apoptotic caspases, mimicking *N*. *gonorrhoeae* OMVs (Fig 10D). In contrast, *E*. *coli* OMVs (*Ec* OMVs) that lacked PorB only caused low rates of macrophage death and activation of caspase-3/7 over time (Fig 10C and 10D). Together, this demonstrates that *N*. *gonorrhoeae* utilizes OMVs to traffic PorB to host cells and to induce mitochondria-mediated apoptosis in macrophages.

## Discussion

The human cervix is primarily patrolled by a large number of resident and recruited macrophages [33]. Despite this, cervical biopsies from gonorrhoea patients highlighted the ability of *N*. *gonorrhoeae* to evade innate immune attack and to replicate extracellularly [34]. *N*. *gonorrhoeae* but not commensal *Neisseria* activate macrophage apoptotic factors independent of bacterial internalization or viability [4]. Whether this depends on secreted membrane vesicles discovered over 40 years ago and known to be highly abundant in gonorrhea patients [34], has not been addressed. Here, we have now shown that *N*. *gonorrhoeae* outer membrane vesicles (OMVs) are sufficient to induce loss of mitochondria membrane potential and apoptotic cell death in *ex vivo* macrophages. We further demonstrated that OMVs contained the virulence factor, PorB, and that the OMVs enabled targeting of PorB to mitochondria, caused loss of mitochondrial integrity, cytochrome *c* release and activation of apoptotic caspases.

OMVs have emerged as a major secretion pathway in bacteria. Typically, vesicles range from 20 to 200 nm in diameter and originate from the bacterial outer membrane due to lipid and/or protein-mediated mechanisms [25]. As a result, OMVs share proteins with the outer membrane. In some cases, OMVs can contain significant cytoplasmic content [29, 35]**,** which may be associated with vesicle formation after explosive cell lysis as observed in *Pseudomonas aeruginosa* [36]. Analysis of *N*. *gonorrhoeae* by transmission and scanning electron microscopy, however, supports the view that vesicles are generated and released form the bacterial cell surface. Indeed, by using stringent purification procedures, we have now shown that OMVs are devoid of cellular fragments and the majority of cytoplasmic proteins. Many of these proteins may therefore not be targeted by OMVs to host cells, as has been suggested previously [29]. A direct comparison of the proteome from highly purified *N*. *gonorrhoeae* OMVs and outer membranes indicated that several proteins are excluded from vesicles, whereas others may specifically be enriched, suggesting that formation of OMVs is a selective packaging event rather than merely pinching of random sections of outer membranes. This is in agreement with other recent proteomic studies of purified OMVs from a diverse group of Gram-negative bacteria [37]. Thus, by using stringent purification methods and comprehensive comparative analysis of proteins, we identified the core *N*. *gonorrhoeae* OMV proteome containing 110 proteins that are largely derived from the outer membrane.

The most abundant protein of OMVs was PorB, which comprised 35% of the total vesicle proteome based on MS/MS spectral counting. PorB is essential for the uptake of small nutrient molecules into *N*. *gonorrhoeae*, and is directly associated with pathogenesis as it can translocate into host cells and target mitochondria [5, 6, 9, 15]. In this study, we have demonstrated that OMVs enable trafficking of PorB from bacteria to mitochondria. Firstly, we were able to detect mitochondria-associated PorB after exposure of macrophages with highly purified OMVs. Secondly, co-incubation of macrophages with *N*. *gonorrhoeae* in transwells that physically separated both cells still enabled PorB trafficking to host cells. Thirdly, delivery of PorB to macrophages was compromised in the absence of sufficient OMV biogenesis. This suggests an orchestrated mechanism by which *N*. *gonorrhoeae* actively induces OMV formation to deliver PorB, and likely many other membrane proteins, to host cells. The underlying mechanism that governs OMV biogenesis remains incompletely described. So far, several genetic mutants in other bacterial species have been identified that show increased or reduced OMV biogenesis [23, 24]. In part, proteins that interfere with the ability of the outer membrane to vesiculate either promote the interaction with the peptidoglycan layer, regulate lipid biosynthesis or alter the membrane lipid composition. Comparatively little is known about the mechanism of OMV biogenesis in *N*. *gonorrhoeae*. Here, we identified that NGFG_01788 positively affected vesiculation. NGFG_01788 may regulate the interaction between the peptidoglycan layer and the outer membrane via its LysM domain. This is supported by a recent study, which identified NGFG_1788 to bind peptidoglycan and to associate strongly with the outer membrane via interactions with PilQ [38]. Both, NGFG_01788 and PilQ, were present in purified OMVs with similar abundance. Unlike PilQ mutants, loss of NGFG_01788 caused the formation of outer membrane protrusion, in agreement with the notion that interactions with the peptidoglycan control outer membrane stability, integrity and protein transport [38]. Whether the reduced vesiculation rate in the absence of NGFG_01788 is a consequence of these membrane protrusion or contributes to surplus outer membrane mass and/or altered membrane properties warrants further investigations.

**Fig 11:**
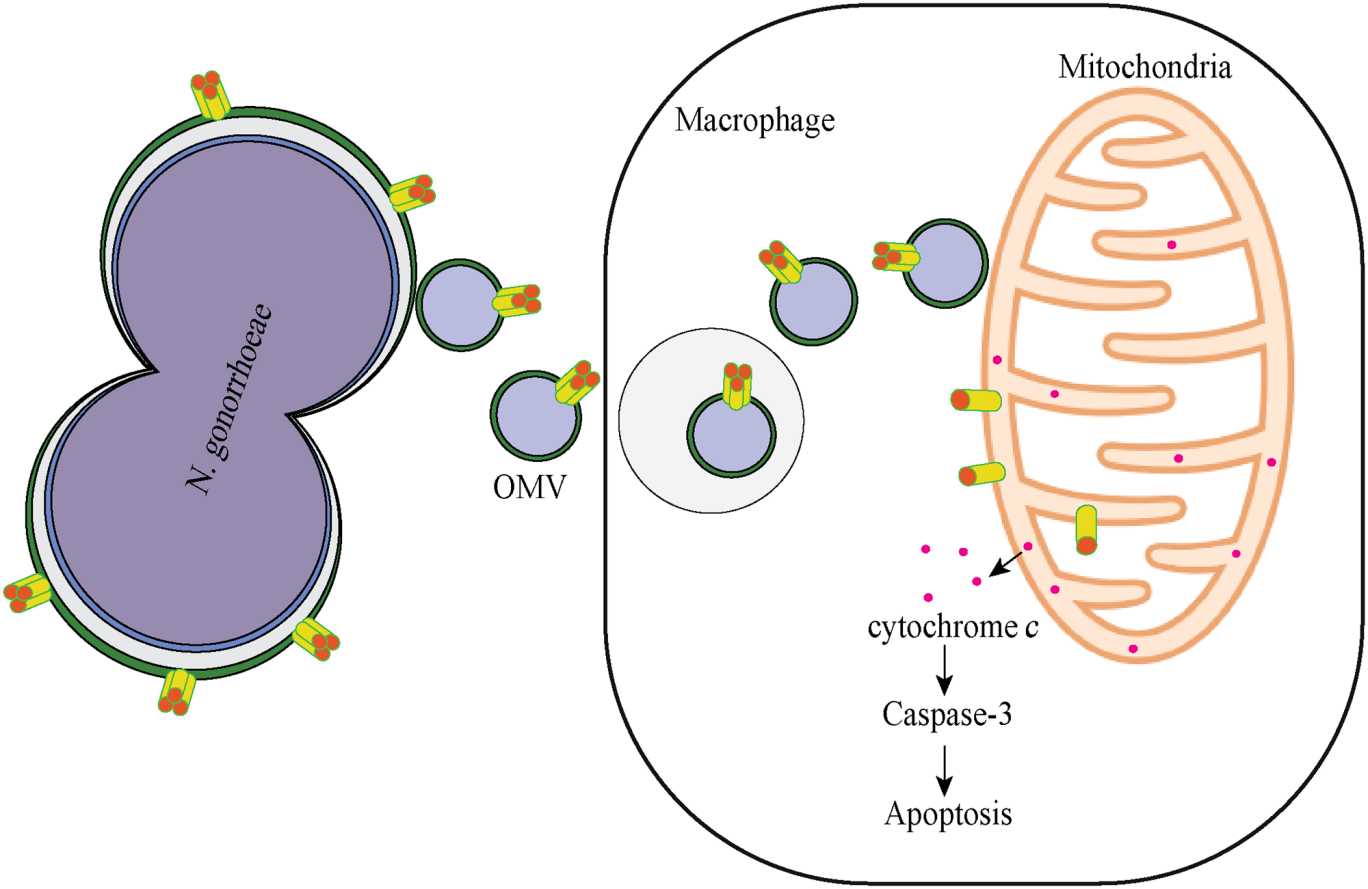
Schematic model of PorB delivery to mitochondria by OMVs. PorB is expressed in the outer membrane of *N*. *gonorrhoeae* as a trimeric complex. PorB is also present in a similar complex in OMVs. Macrophages sense and take up OMVs, although the mechanism remains to be elucidated. Intact *N*. *gonorrhoeae* OMVs containing PorB are released into the cytosol, suggesting escape from endo-lysosomal compartments. Cytosolic OMVs in close proximity to mitochondria enable transport of PorB and other OMV proteins such as BamA to mitochondrial membranes, including outer and inner membranes. Mitochondria-associated PorB is largely present as monomeric protein. PorB targeting of mitochondria induces cytochrome *c* release, the activation of caspase-3, plasma membrane blebbing and macrophage cell death.

Professional phagocytes, including macrophages, but also epithelial cells readily take up OMVs via endocytic pathways [39]. The fate of intracellular OMVs remains less clear as their localization is based on the detection of specific cargo proteins and lipids, which can dissociate from vesicles inside host cells. In epithelial cells, OMVs are thought to be targeted to lysosomes for degradation [25]. In macrophages, OMV-derived LPS was detected within the cytosol, rather than lysosomes, suggesting that OMVs may escape endosomes [40]. We detected *N*. *gonorrhoeae* OMVs that appeared cytosolic based on the absence of surrounding host membranes 24 hours post treatment. It is possible that PorB dissociates from cytosolic OMVs, which would enable translocation into mitochondria using host import machinery, a phenomenon that can be mimicked experimentally [6, 15]. However, PorB was largely present as ß-barrel protein complex in OMVs and in this native state, a machinery which can only import unfolded proteins may not be readily accessible to the mitochondrial import machinery. As a potential alternative explanation, OMVs may directly associate with mitochondria to deliver folded PorB. Consistent with this, mitochondria-associated PorB was frequently observed in clusters, which may arise from direct contact of vesicles with mitochondrial membranes. Electron microscopy also indicated a close proximity of OMVs with mitochondria. Furthermore, incubation of OMVs with isolated mitochondria enriched fractions resulted in the association of OMV-derived PorB with these fractions. It remains unclear whether a close association is sufficient to transfer membrane proteins to mitochondria or whether membrane fusion occurs. The latter has been experimentally observed using *Legionella*-derived OMVs and model host membranes [41]. Membrane fusion could potentially also explain the BamA targeting to mitochondria after OMV exposure *in vivo* and *in vitro*. Future efforts are directed in validating the current model whereby *N*. *gonorrhoeae* OMVs are endocytosed by macrophages, escape endo-lysosomal compartments and directly deliver membrane proteins to mitochondria, perhaps independently of previously identified mitochondrial import pathways (Fig 11).

PorB shares several features with the host mitochondria porin, VDAC. Similar to VDAC, PorB targets the outer mitochondrial membrane and affects cell death signaling by disrupting mitochondria membrane potential (ΔΨm) [5, 6, 9]. How PorB affects apoptosis remains controversial. PorB itself was thought to be insufficient to induce apoptosis and was dependent on additional host pro-death factors [7, 8]. Also, not all PorB homologs induce cell death, as *N*. *meningitidis* PorB targets mitochondria to prevent apoptosis [10, 17]. Of note, these studies used high expression levels, tagged or detergent isolated PorB, and/or largescale protein transfection to expose host cells to PorB. Here, we demonstrated that PorB containing OMVs from *N*. *gonorrhoeae* caused the loss of mitochondrial membrane potential, the release of cytochrome *c*, activation of apoptotic caspases and macrophage cell death over time. Using time-lapse imaging of single cells, *N*. *gonorrhoeae* OMV exposure resulted in loss of mitochondrial membrane potential within 10 hours. Activation of caspase-3 and macrophage death occurred only after 15 hours, which also coincided with detectable levels of PorB in mitochondria. As observed in other studies [16], we were unable to genetically delete PorB in *N*. *gonorrhoeae* or inducibly shut down its expression. However, inducible expression of native PorB in HeLa cells resulted in mitochondria targeting and fragmentation and activation of caspase-3. Furthermore, *E*. *coli* OMVs containing gonococcal PorB showed increased ability to activate host caspases and kill macrophages, comparable to *N*. *gonorrhoeae* OMVs. This suggests that PorB is a major factor of *N*. *gonorrhoeae* OMVs that induces apoptosis. Previously, ectopically expressed tagged PorB formed protein complexes in mitochondria resulting in loss of membrane potential [6, 9], although mitochondria localized monomers have also been identified [32]. While PorB is present as oligomeric complex in OMVs derived from *N*. *gonorrhoeae*, PorB was exclusively monomeric after association with mitochondria. It will be important to extend a similar analysis to PorB expressing *E*. *coli* as well as other *Neisseria* species to provide further evidence that monomeric rather than oligomeric porins cause apoptosis. In support of this, we observed monomeric PorB in mitochondria after ectopically expressing the native porin, which was sufficient to induce apoptosis. Presumably, monomeric PorB is unable to form pores to damage mitochondria, suggesting that it functions differently in mitochondria than in bacterial outer membranes. Whether this includes the interaction with mitochondrial proteins either in the outer or inner membrane to form transit and/or unstable complex awaits further investigation.

Other pathogens target mitochondria during infections. The *E*. *coli* pore forming protein hemolysin (EHEC-Hly) induced mitochondria-mediated apoptosis, but only 26 hours after treatment of cells with OMVs [25]. Besides PorB and hemolysin, other virulence factors have emerged to cause cell death after delivery via OMVs, including shigella toxin 2a, cytolethal destending toxin V and flagellin from enterhemorrhagic *E*. *coli* and OmpA from Acinetobacter [27, 42, 43]. In contrast to these studies, PorB is the first membrane protein identified to target mitochondria via OMVs to induce apoptosis. It is likely that other porins utilize OMVs for mitochondria targeting, as *V*. *cholera* porin OmpU and *P*. *aeruginosa* porin are present on OMVs. This suggests that OMVs promote bacterial infections by altering host cell responses, as recently shown during *Legionella*-macrophage interactions [44]. It is tempting to speculate that OMV-delivered PorB targets and eliminates macrophages to the benefit of the pathogen. *N*. *gonorrhoeae* also escapes neutrophil attack, despite their accumulation at the infections site. Given that neutrophils show reduced levels of apoptosis in *N*. *gonorrhoeae* infections [45, 46], it will be of interest to determine the effects of OMVs on these host cells.

## Materials and methods

### Bacterial strains and growth conditions

*Neisseria gonorrhoeae* FA1090 and MS11-A strains were cultured as described previously [47]. Unless otherwise indicated, OMVs were purified from the highly virulent *N*. *gonorrhoeae* MS11-A strain [48]. The deletion of NGFG_01788 in the MS11-A strain was carried out as described previously [49]. Briefly, 500 bp upstream and downstream of the open reading frame were synthesized as Gblocks (IDT), including a BamHI site between them. The Gblocks were A-tailed and cloned into pGEM-T Easy Vector system (Promega). Next, the kanamycin resistance cassette (flanked by flipase FRT sites) was amplified from the plasmid pKD4 and cloned into the BamHI site between up- and downstream fragments. A *Neisseria* DNA uptake sequence (ATGCCGTCTGAA) was included upstream of the kanamycin resistance cassette. The resulting plasmid was linearized with the SacI restriction enzyme and added dropwise onto plates (20–40 ng/mL). 3–4 piliated colonies were streaked across the DNA. After 24 h, colonies on the DNA-spotted area were swabbed and transferred to GC-kanamycin plates. Homologous recombination was confirmed by PCR using primers to determine correct targeting of the kanamycin resistant cassette and loss of NGFG_01788.

### Ethics statement

Animal experiments were performed in accordance with the National Health and Medical Research Council Australian Code of Practice for the Care and Use of Animals and were approved by the Monash University Animal Ethics Committee (approval no. MARP2016/099). All mice were maintained under specific pathogen-free conditions.

### Generation of murine bone marrow derived macrophages (BMDMs)

BMDMs were generated from the femurs and tibias of 6–8 weeks-old wild type C57BL/6 mice (obtained from Monash Animal Research Platform, Monash University) as described previously [31]. Briefly, bone marrow was flushed from the bones using 26 G x ½ in needle and 10 mL of BMDMs culture medium, comprising RPMI-1640 media supplemented with 15% FBS (Serena), 15% L929-cell- conditioned medium (containing macrophage colony-stimulating factor), 25 mM HEPES and 100 U/mL Penicillin/Streptomycin (Sigma). Cells were placed in 25 cm^2^ tissue culture-treated flasks overnight at 37 °C, 5% CO_2_. The following day non-adherent cells were transferred to bacteriological petri dishes and cultured for 7 days, at 37 °C, 5% CO_2_. For OMV treatment, BMDM were gently scrapped from the petri dishes using a cell scrapper (BD Falcon) and were seeded in tissue culture-treated multi well plates at a density of 2.5 x 10^5^ or 5 x 10^4^ cells/well for relevant assays.

### Isolation and purification of *N*. *gonorrhoeae* secreted outer membrane vesicles (OMVs)

*N*. *gonorrhoeae* were cultured in 450 mL of gonococcal (GC) liquid media supplemented with Deakin modified isovitalex (DMIV) and 0.01M NaHCO_3_ at 37 °C shaking at 200 rpm. After centrifugation (12,000 × g for 12 min at 4 °C) the supernatant was passed through Nitrocellulose MF-Millipore^™^ membrane filters (0.45 μm) to remove any remaining whole cells and cellular debris. Filtered supernatant was then ultracentrifuged to pellet OMVs using a 45-Ti fixed angle rotor and Thermo Scientific SORVALL WX Ultra at (186,000 × g for 3 h at 4 °C). Pelleted OMVs were washed twice with PBS to remove liquid media. This crude OMV preparation was free of any bacterial contaminants and stored at -80 °C. To isolate OMVs with higher purity, crude vesicles preparations were subsequently subjected to OptiPrep density gradient ultracentrifugation whereby crude OMVs were mixed with 65% (w/v) Optiprep to obtain final concentration of 55% (w/v) OptiPrep OMVs suspension. 2 mL of the OptiPrep OMV suspension was then loaded at the bottom of a SW- 40 rotor tube. 1.8 mL of discontinuous OptiPrep gradient [50, 45, 40, 35, 30 & 25% (w/v)] decreasing from bottom to top were spun at 146,000 × g for 16 hours at 4 °C using a SW-40 rotor. 11 equal fractions were collected sequentially from the top of the tube using a Brendel Foxy Jr. gradient fractionator. The protein concentration of each of the fractions were also measured using the BCA method (Thermo Scientific). All collected fractions were subjected to tricarboxylic acid (TCA) precipitation and then analyzed by separation on a 12% (w/v) acrylamide SDS-gel and stained with brilliant colloidal coomassie stain.

### Proteomic characterization of OMV by LC-MS/MS

Crude and purified OMV preparations were subject to mass spectrometry using the FASP method by following the protocol of FASP protein digestion kit (Expedeon). Peptides obtained by tryptic digestion were then mixed with 2-acetonitrile and 0.1% formic acid. Samples were analyzed by LC-MS/MS using a Q Exactive Orbitrap mass spectrometer (Thermo Scientific) or Q Exactive Plus Orbitrap mass spectrometer coupled online with a RSLC nano-HPLC (Ultimate 3000, Thermo Scientific). Spectra were converted to mzML files using MSconvert, facilitated by ProteoWizard software [50], and were searched using X!Tandem [release 2009.10.01.1 LabKey, Insilicos, Institute for Systems Biology, [51]] contained within the Trans-Proteomic Pipeline [v4.6; [52]] with high-resolution k-score against *N*. *gonorrohoeae* FA1090 and MS11 strains were searched from UniProt database supplemented with known contaminants. Reverse sequences (Decoys) informed false positive identification frequency. Search parameters were as follows: precursor ion mass (monoisotopic) and MS/MS tolerance ± 20 ppm with trypsin cleavage allowing for up to 2 missed cleavages. Modifications included fixed Cysteine carbamidomethylation and variable methaionine oxidation and protein N-terminal acetylation. Peptide Prophet (v4.6) was used to curate peptide-spectrum matches of FDR ≤ 1% and assign representative proteins/ protein isoforms/ accessions. To estimate the relative abundance of identified proteins within a sample, we used label-free spectral counting which is based on the number of MS/MS spectra mapped to peptides with high confidence (spectra-peptide matches) to calculate the percentage share of the spectra within a sample [53].

### Bioinformatic analysis

Proteins identified by mass spectrometric analysis were annotated for subcellular localization using four different available online bacterial protein subcellular localization (SCL) predictor tools, PSORTb version 3.0.2 [54], SubLoc v1.0 [55], CELLO 2.5 [56] and PSLpred [57]. The predicted subcellular component was assigned based on majority votes. The identified protein was annotated as unknown subcellular localization in a case where all four tools predicted different subcellular localization for a particular protein. Identified proteins were classified phylogenetically using Clusters of Orthologous (COGs) and functional categories were assigned using WebMGA [58].

### *In-vitro* PorB expression

Tet-On advanced HeLa cells were transiently transfected with pTRE.PorB.IVS.IRES.eGFP, pTRE.Puro.PorB and pTRE.IVS.IRES.eGFP plasmids using Lipofectamine 2000 according to manufacturer’s protocols. Briefly, HeLa cells were seeded onto coverslips in 24/6 well plates to obtain 80–90% confluency (~2.0 using Basal Medium Eagle (BME) growth medium supplemented with 5% (v/v) heat inactivated serum, 5% (w/v) Glutamine and 100 Units/mL Penicillin/Streptomycin. 2 μg of plasmid DNA obtained from Maxi-prep was mixed in 0.5 mL of growth medium without serum. 20 μL of Lipofectamine 2000 (Invitrogen) was diluted in 0.5 mL of growth medium without serum. Diluted DNA and Lipofectamine, was mixed (ratio 1:5) and incubated at room temperature for 20 min and added to each well of monolayer HeLa cells. After 5 hours of the transfection media was changed with fresh media containing FBS, L-glutamine and Penicillin/streptomycin. 24 hours of post transfection doxycycline was added and the gene expression was monitored using fluorescence microscopy and immunoblot analysis.

### PorB expressing *E*. *coli* OMVs

Gonococcal *porB* was amplified by PCR from genomic DNA isolated from *N*. *gonorrhoeae* MS11 strain using primers IDH117 (ACTGACCATGGgtGATGTCACCCTGTACGGTGCCATC) and IDH118 (GAATTCTCGAGTTAGAATTTGTGGCGCAGAACGACGG). The product was cloned into the *Nco*I and *Xho*I sites of a modified pET20b vector with a pelB signal sequence and 10XHis tag to generate pET-PorB [59]. IPTG was used to induce the expression in *E*. *coli*, which was confirmed by coomassie stain and immunoblot analysis. The OMVs secreted by PorB expressing *E*. *coli* were isolated and purified as discussed above.

### Bacteria-macrophage interactions in transwells

For the trafficking of OMVs from bacterial cells to BMDMs, 1 × 10^5^ BMDM cells were seeded onto coverslips in a 24 well plate. *N*. *gonorrhoeae* MS11-A (wild-type) and *ΔNGFG_01788* were placed in a transwell (Corning #3470) of pore size 0.4 μm at MOI of 1: 20 for 24 hrs. No bacteria were recovered from the BMDM containing well. Migration of OMVs to BMDMs were analyzed by immunofluorescence assay.

### Immunofluorescence analysis (IFA)

BMDMs (1 × 10^5^ cells) were seeded onto sterile glass cover slips of 18 × 18 mm dimension (MENZEL-GLASER, Thermo Scientific) in 24-well plates or directly in 8-well tissue culture chambers (Sarstedt) and treated with *N*. *gonorrhoeae* isolated OMVs at concentrations of 20–50 μg/mL or as indicated otherwise. Cells were fixed in ice-cold 4% (w/v) paraformaldehyde (PFA) for 10 min, washed three times with PBS and quenched with 0.1% (w/v) NaBH_4_ (Sodium tetra borohydride, Sigma) for 10 min. Cells were then permeabilized with 0.1–0.2% Triton-X 100 in PBS on ice for 5 min and blocked with PBS containing 3% (w/v) BSA and 0.1% (w/v) sodium azide in PBS overnight at 4 °C. After blocking, the cells were incubated with anti-PorB and anti-Tom 20 (abcam-#56783). After three washes with ice cold PBS, the cells were then incubated with corresponding anti-rabbit/mouse-coupled with Alexa-Fluors (488 and 594) (Life Technologies) containing 0.1 μg/mL of DAPI (Sigma) for 1 hour. After three washes in PBS, coverslips were mounted on glass slide of dimension 76 × 26 mm (MENZEL-GLASER, Thermo Scientific) in fluorescence mounting medium (DAKO) and imaged on Nikon Inverted Confocal laser scanning microscope using 100X/ 60X objective 0.8 numerical aperture (NA). More than 50 individual cells/group from three biological repeats were analyzed manually in Fiji.

### Super-resolution microscopy

Super-resolution images were recorded via dSTORM (direct stochastic optical reconstruction) using an Olympus IX-71 microscope and UPlanSApo UIS2 oil-immersion 100 × NA 1.4 objective, 1.6 × magnification changer, Toptica 488 nm laser (200 mW), Gem 561 nm laser (500 mW) and Oxxius 638 nm laser (150 mW), suitable Olympus fluorescence filter cubes and an Andor iXon Ultra 897 High Speed EMCCD camera with single photon sensitivity for single molecule detection. The final excitation steering mirror and beam expansion lenses are mounted on a translation stage for free adjustment of the TIRF (total internal reflection fluorescence) angle. The system was operated at a TIRF angle appropriate for the respective sample to concentrate excitation power and reduce background fluorescence. Samples were mounted on a manual xy translation stage to minimise sample drift. For Alexa-Fluor 647 super-resolution images, the samples were illuminated continuously with 70 mW, 638 nm laser power at the appropriate TIRF angle. After an initial pumping period of < 30 sec to drive dyes into the dark state, single molecule blinking time series were acquired for 10,000 frames at an exposure time of 20 milliseconds (unless otherwise stated). Raw image pixel size with 100 × objective and 1.6 × magnifier engaged is 100 nm. For dual colour experiments, images were acquired sequentially. First, the Alexa-Fluor 647 super-resolution image was recorded with the settings mentioned above. Subsequently Alexa Fluor 568 was imaged after an initial pumping period of approximately 1 min at 180 mW, 561 nm laser power. For imaging, the laser power was reduced to 120 mW and single molecule blinking time series were acquired for 10,000 frames at an exposure time of 20 milliseconds (unless otherwise stated). The acquired data was reconstructed to super-resolution images with a pixel size of 10 nm using the open-source software rapid STORM 3.3.1 [60]. Blinks with a local signal-to-noise ratio (SNR) < 80 were discarded. Images were first colour coded for temporal appearance of blinks to detect sample drift, then (if applicable) corrected for drift using the linear drift correction available in rapid STORM and exported as 8-bit greyscale images for further processing.

### 3D STORM dual colour single molecule image acquisition and processing

3D dual colour single molecule super-resolution images were acquired using the Vutara SR 350 system (Bruker) using similar dual-colour buffer consisting [5% (w/v) glucose, 40 μg/mL catalase, 0.5 mg/mL glucose oxidase, 10 mM MEA (Cysteamine Hydrochloride) pH 8.0 in PBS]. Multi-colour fluorescent tetra-spec beads were used for point spread function calibration. The system was inbuilt with imaging laser lines (488 nm, 560 nm, 640 nm and 750 nm, all 1 W laser power attenuated to 300 mW), one activation laser (405 nm, 100 mW), 60 × oil immersion objective, sCMOS Hamatasu Camera Orca Flash 4.0 and quad field module (Orange/Red) for 3D imaging using the biplane approach. Images were acquired by pumping all the dye into the dark state at a laser power of 11 kW/cm^2^. Individual dye molecules were reactivated with low amounts of 405 nm laser light (25 W/cm^2^), over the imaging time to keep the number of detected blinks at approximately the same level. 100 frames were taken for each z-slice (2–3 z-steps), with 20–25 repeats at an exposure time of 10–20 milliseconds. 3D super-resolution image reconstruction was performed using the Vutara 350 SRX Software, where the background threshold was set to 10, a confidence value of 0.8 was used and the particle size was set to 50 nm diameter. Images are displayed with colour relevant to z-depth.

### Transmission electron microscopy

20 μL of crude and purified OMV preparation (approx. 50 μg/mL) were fixed in 2.5% glutaraldehyde and placed on carbon-coated 300- mesh nickel grids (3.05 mm diameter: 0.4 × 2 mm single slot) washed in poly-_*L*_-lysine. After two washes in PBS and one wash in distilled water, the grids were then stained with 1% (w/v) uranyl acetate for 5 min. Methyl-Cellulose in 1% (w/v) uranyl acetate was used to form the protective layers of the grids overnight. The grids were then viewed with a HITACHI H-7500 electron microscope operated at 80 kV. The images were taken with a GATAN Multiscan 791 CCD camera. Liquid cultures of *N*. *gonorrhoea* were grown to the indicated growth phase, as described above. 1 mL of harvested bacterial cells were then washed twice with 0.1M Sodium cacodylate buffer solution and fixed with 2% (w/v) glutaraldehyde in 0.1 M EM quality phosphate buffer solution (pH 7.4) for 30 min. Cells were then subsequently washed twice with 0.1 M Sodium cacodylate buffer and dehydrated in a series of graded ethanol-dilutions [50, 60, 70, 80, 90 and 100% (v/v)] for 3 min each. Finally, specimens were air-dried in a desiccator overnight and observed under FEI Nova Nano Scanning electron microscope 450.

### Gelatine embedding and immunogold labelling

OMV and bacterial cells were washed 3 times for 5 min each with 0.1 M EM quality Phosphate buffer (PB) and then incubated in 0.1 M PB containing 0.15% glycine for 5 min. Next the samples were then incubated in 1% gelatine in 0.1 M PB for 15 min at room temperature. The cells were then scrapped into a tube and spin down to pellet. The pelleted cells were replaced with 2% gelatine and incubated at room temperature for 30 min at 37 °C. This was repeated again with 6% and, finally with 12% gelatine. The tubes were then transferred to ice for gelatine solidification. The tubes were then cut and the gelatine samples were placed in 2.3 M sucrose. The gelatine samples were cut into large section approx. 1 mm thick and placed in a tube with 2.3 M sucrose. Sucrose was allowed to infuse on a rotator at 4 °C overnight. Smaller sections were finally cut and mounted on cryopin for storage in liquid nitrogen. Ultrathin frozen sections of 60–90 nm were prepared using Leica Ultracut UC7 FCS. The sections were then probed with anti-sera raised against PorB followed by corresponding Protein-A Gold (10 nm gold particle). Staining with Protein-A Gold alone, without primary antibody, was included as control for specificity of the immunogold staining.

### Transmission electron microscopy of ultrathin section for bacterial membrane integrity and vesiculating whole bacterial cells

Whole bacterial cells grown to mid-log phase were harvested at (4,000 × g for 5 min) and the cell pellet was washed twice with PBS. The cells were then fixed in 2.5% (w/v) glutaraldegyde in 0.1 M sodium cacodylate buffer, pH 7.2, for 2 hours at room temperature. After fixation, the samples were rinsed three times for ten min each with 0.1 M sodium cacodylate buffer and incubated in post fixation solution (1% osmium tetraoxide, 1.5% potassium ferricyanide in 0.065 M sodium cacodylate buffer, pH 7.4). The samples were then washed with distilled water five times, 10 min each and serially dehydrated using increasing concentrations (80%, 90%, 95% and 100%) of ethanol. After dehydration the samples were rinsed in epon araldite resin, with increasing concentrations (3:1, 2:1, 1:1, 1:2 and 1:3) of propylene oxide:epon in a Bio-wave pro (Pelco) with programmed heating. Finally, samples were left in 100% epon araldite resin for 48 h. Embedded bacterial cells in resin were cut into 50 to 70 nm thin-sections of using a Leica Ultracut UC7 FCS. Sections were then stained with 2.5% uranyl acetate for 15 min followed by 3 min in Reynold’s lead citrate and imaged using HITACHI H-7500 electron microscope operated at 80 kV using a Gatan camera and Digital micrograph 1.71.38 (Gatan Incorporated).

### Cytosolic and mitochondrial fraction isolation from BMDM and immuno blot analysis for cytochrome *c* release

Cytosolic and mitochondrial fraction from OMV, PBS and STS treated BMDMs were isolated as described previously but with some modifications [61]. Briefly, cells were washed twice with PBS and harvested by scraping. Harvested cells were resuspended in ice cold isotonic solution (20 mM HEPES pH 7.5, 220 mM mannitol, 70 mM sucrose, 1 mM EDTA and complete EDTA free protease inhibitor cocktail (Roche). Cells were lysed by repeated syringing through a 23-gauge needle on ice. Cell lysate were then centrifuged (1,000 × g for 15 min at 4 °C) to pellet down nuclei and unbroken cells. The supernatant was then centrifuged (16,000 × g for 60 min at 4 °C) to collect the pelleted mitochondrial fraction and supernatant as the cytosolic fraction. The cytosolic fraction was also checked under a microscope for any cellular fraction, and centrifuged (16,000 × g for 15 min at 4 °C) again to remove any cellular debris. The protein concentration of the isolated mitochondrial and cytosolic fraction was measured by BCA assay (Thermo Scientific). The cytosolic and mitochondrial fraction were analyzed following separation by SDS-PAGE and, immunoblot analysis using anti-cytochrome *c* (Abcam #13575), anti-Tim23 (BD #611222).

### OMV fusion with mitochondrial fraction assay

Mitochondrial fraction from bone marrow derived macrophages were isolated as described above and suspended in isotonic solution. Two tubes containing isolated mitochondrial fractions in isotonic buffer were treated with PBS and OMV and left at room temperature for 3 hours. After incubation, the tubes were spined down at 16× 000 g for 30 min. The control tube containing OMV in isotonic buffer was also spined to see if 16× 000 g is sufficient to pellet down OMVs. The supernatant and pelleted fraction were separated and analyzed by immunoblot analysis by probing for PorB and Tim23.

### MTT assay

BMDMs were seeded in 96-well tissue culture plates at 1 × 10^5^ cells/well and incubated at 37 °C, 5% CO_2_ for overnight. The cells were then treated with RPMI-1640 culture medium containing OMVs at the indicated concentrations and time. After treatment, the medium was replaced with a medium containing 0.5 mg/mL MTT reagent (Thermo Scientific), and incubated at 37 °C for 4 hours. The medium was then discarded and the reaction was stopped by adding freshly prepared solubilizing solution [10% (w/v) SDS and 0.01 M HCl, pH 2.0] into each well, followed by incubation at 37 °C for 10 min. After incubation, the plate was gently shaken for complete dissolution of tetrazolium salts. 200 μL of the resulting solution was transferred onto new 96-well plate and the absorption of formazan colour formed was measured at 560 nm using Tecan F200 plate reader (DKHS). The absorption of PBS treated cells was obtained as the relative percentage of viable cells. The data of percentage cell viability were analyzed in Excel and GraphPad Prism.

### Live-cell imaging to determine macrophage viability, ΔΨm and activation of caspase-3

Cellular morphology, cell death, loss of mitochondrial membrane potential (ΔΨm) and activation of caspase-3/7 were monitored as recently described [31] with some modifications. Briefly, BMDMs were seeded in 96-well tissue culture plate at a density of 5 × 10^4^ cells/well. Prior to OMV treatment, cells were stained with 1 μM Cell Tracker Green (CTG, Life Technologies) for 30 min in serum-free RPMI-1640. Medium was then replaced with culture medium containing 600 nM Draq7 (Abcam), 50 nM TMRM (Life Technologies) or CellEvent Caspase-3/7 Green Detection Reagent (Thermo Scientific) (1 drop/mL), with, or without specific inhibitors for 30 min. The cells were then treated with OMVs at a concentration of 50 μg/mL, or at the indicated concentration prepared from three independent cultures. In some experiments, cells were exposed to 500 nM ABT-737 (Abbvie), 50 nM staurosporine (Sigma), 1 μg/mL cyclohexamide (Sigma) and 20 μM pan-caspase inhibitor Q-VD-OPh (R&D System Biology) and imaged with a Leica AF6000 LX epi-fluorescence microscope. The microscope was equipped with an incubator chamber set at 37 °C and 5% CO_2_ and an inverted, fully motorized stage driven by Leica Advanced suite application software. Time-lapse images were acquired with bright field, GFP, TxRed and Y5 filters every 30 mins for up to 48 h using a 10 ×/ 0.8 –NA objective. To determine the percentage of dead cells and cells with reduced ΔΨm, images were analyzed using ImageJ and Metamorph (Molecular Devices) using a custom-made journal suite incorporating the count nuclei function to segment and count the number of the TMRM, caspase-3/7 and Draq7-positive cells based on CTG stained cells. The data were analyzed in Excel and GraphPad Prism.

### Semi-native-gel electrophoresis

Sonicated bacterial membrane and OMVs were solubilized by resuspension in 1×-loading buffer (0.05 to 1% (w/v) SDS (sodium dodecyl-sulfate), 30 mM Tris-HCl (pH 6.8), 5% (v/v) glycerol and bromophenol blue as migration indicator. For heat treatment, samples were further incubated at 95 °C for 10 min before loading. Proteins were separated by semi-native PAGE as previously described [15]. The proteins were transferred onto PVDF membrane and detected by antibodies (anti-PorB) using enhanced chemi-luminescence imaging methods.

### Immunoblot analysis

After separation by SDS-PAGE, gels were wet transferred at 4 °C onto a nitrocellulose membrane, 0.45 μm (Millipore) and 0.2 μm (Biorad), at a maximum of 100 V, 350 mA, for 60 min, in 0.5% transfer buffer (380 mM glycine, 202 mM Tris, 0.02 (w/v) SDS and 20% (v/v) methanol). Ponceau staining (2.5% (w/v) Ponceau, 5% (v/v) acetic acid) was used to confirm protein transfer efficacy. Membranes were then incubated with blocking buffer TBS-T containing 3% (w/v) skim milk or 3% (w/v) BSA (Sigma)) for 1 h at room temperature. TBS-T contained 0.2% Tween-20, 137 mM Nacl, 2.7 mM KCl, 25 mM Tris, pH 7.4. Membranes were then probed with primary antibodies resuspended in blocking buffer overnight, at 4 °C. After primary antibody incubation, the excess of antibody was washed-off by washing membrane with TBS-T three times 5 min each. After washing, the membrane was then probed with appropriate secondary antibodies conjugated to horseradish peroxidase (HRP). Membranes were developed with the ECL reagent, Clarity ECL (Biorad), Super signal West Femto ECL (Thermo Scientific), or Amersham ECL Prime (GE Healthcare) before exposure to film (Kodak). Images were then scanned and processed with Fiji and Adobe Illustrator.

## Supporting information

S1 Fig OMVs deliver PorB to human macrophages mitochondria. Human THP-1 macrophages were incubated with *N*. *gonorrhoeae* OMVs and PorB and Tom20 localization was determined by immunofluorescence analysis after 24 hours.
(TIF)

S2 Fig NGFG_01788 promotes OMV biogenesis. (A) Purified OMVs and whole bacterial lysates (WBL) from equal numbers of wild type (WT) and ΔNGFG_01788 deletion mutant were stained with coomassie after gel-electrophoresis. PorB is indicated based on immune blot analysis. NR indicates not-relevant lane. (B) Total protein content of purified OMVs from WT and ΔNGFG_01788 deletion mutant was determined by the BCA assay relative to bacterial numbers (OD 600 nm). Mean and SD from three independent experiments.
(TIF)

S3 Fig *N. gonorrhoeae* OMVs induce sequential loss of mitochondrial health, caspase activation and cell death in macrophages. BMDMs were labelled with TMRM (red), exposed to OMVs and incubated with caspase-3/7 specific fluorogenic substrate (green) and Draq7 (blue). Time-lapse images are shown from indicated time frames. Arrow indicates a macrophage that sequentially lost TMRM signal, activates caspase-3/7 and stained positive for Draq7. Scale bar = 100 μm.
(TIF)

S4 Fig Ectopically expressed PorB targets mitochondria and induces apoptosis. Tet-On advanced Hela cells were transiently transfected with pTRE-Tight response plasmids (pTRE.PorB.IVS.IRES.eGFP and pTRE.Puro.PorB). (A) Doxycycline dependent ectopically expressed PorB (green) colocalized with Tom20 (red) in a time dependent manner, causing the loss of mitochondrial network. (B) Doxycycline induced expression of PorB caused cleavage of caspase-3 (17 kDa) as detected by immunoblot analysis. Tubulin staining is shown as a loading control. (C) Semi-native gel electrophoresis shows heat sensitive PorB complex formation in OMVs but monomeric PorB in HeLa cells after doxycycline induction. Tim23 and F_1-_β are shown as loading controls.
(TIF)

S1 Video Live cell imaging of OMV treated macrophages. BMDMs were labelled with TMRM (red), exposed to OMVs and incubated with Draq7 (blue) and caspase-3/7 fluorogenic substrate (green). Time-lapse movie showing bright field and fluorescent images every 30 minutes for 48 hours.
(AVI)

S2 Video Live cell imaging of PBS treated macrophages. BMDMs were labelled with TMRM (red), treated with PBS and incubated with Draq7 (blue) and caspase-3/7 fluorogenic substrate (green). Time-lapse movie showing bright field and fluorescent images every 30 minutes for 48 hours.
(AVI)

S1 Table The proteome of purified and crude *N. gonorrhoeae* OMVs. *N*. *gonorrhoeae* MS11-A OMVs were isolated from culture supernatants (crude) or further purified by gradient ultracentrifugation (pure) and their proteome determined by LC-MS/MS. The complete list shows all identified proteins and their relative abundance.
(XLSX)

S2 Table Subcellular annotation of the OMV proteome. Proteins identified in *N*. *gonorrhoeae* OMVs (crude and pure) were analyzed by subcellular localization predictor tools and their predicted localization was assigned based on the majority of votes.
(XLSX)
