# Supplementary figures and images for: Outer membrane vesicles from Neisseria gonorrhoeae target PorB to mitochondria and induce apoptosis
Source: PLoS Pathog. 2018 Mar 30;14(3):e1006945. doi: 10.1371/journal.ppat.1006945 (PMC5877877; doi:10.1371/journal.ppat.1006945)

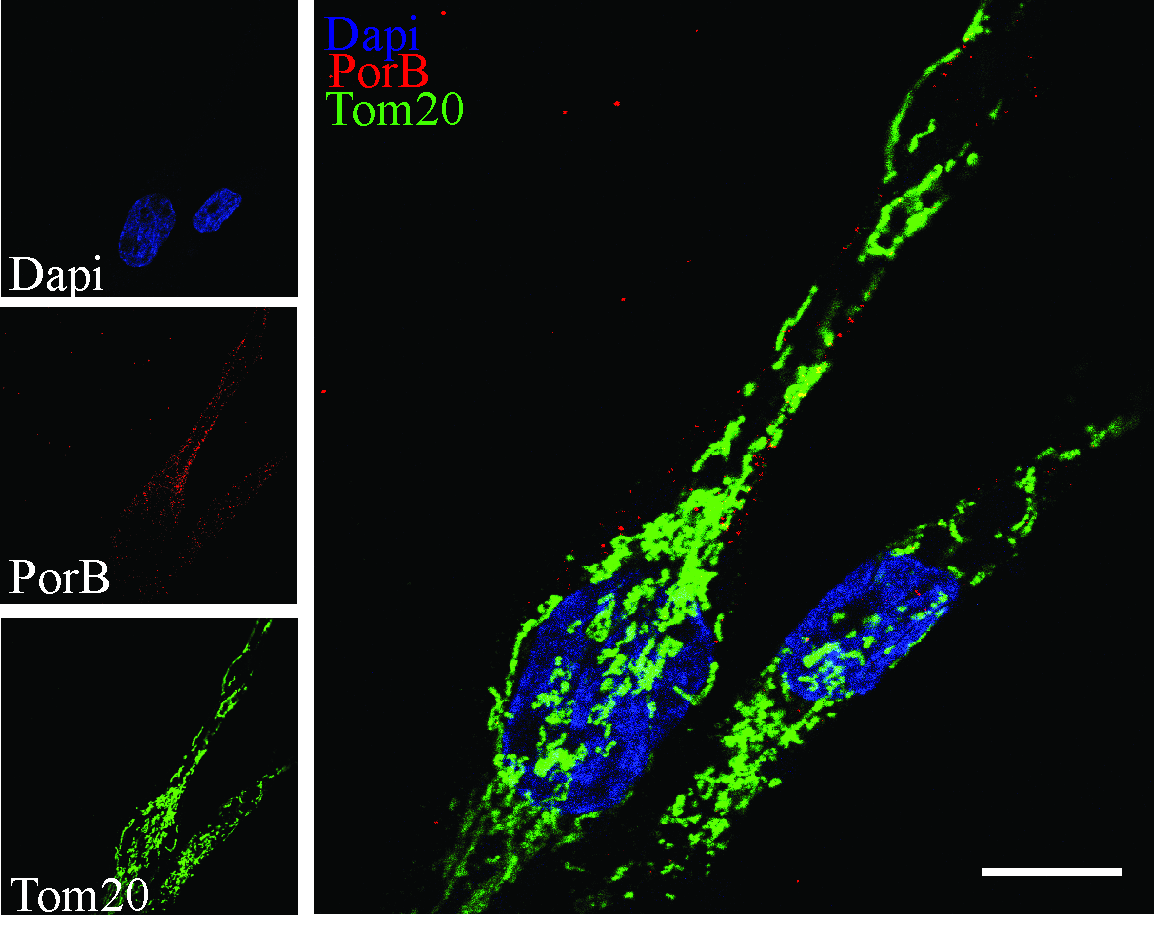

Supplement: S1 Fig — Human THP-1 macrophages were incubated with N. gonorrhoeae OMVs and PorB and Tom20 localization was determined by immunofluorescence analysis after 24 hours. (TIF) [file ppat.1006945.s001.tif]

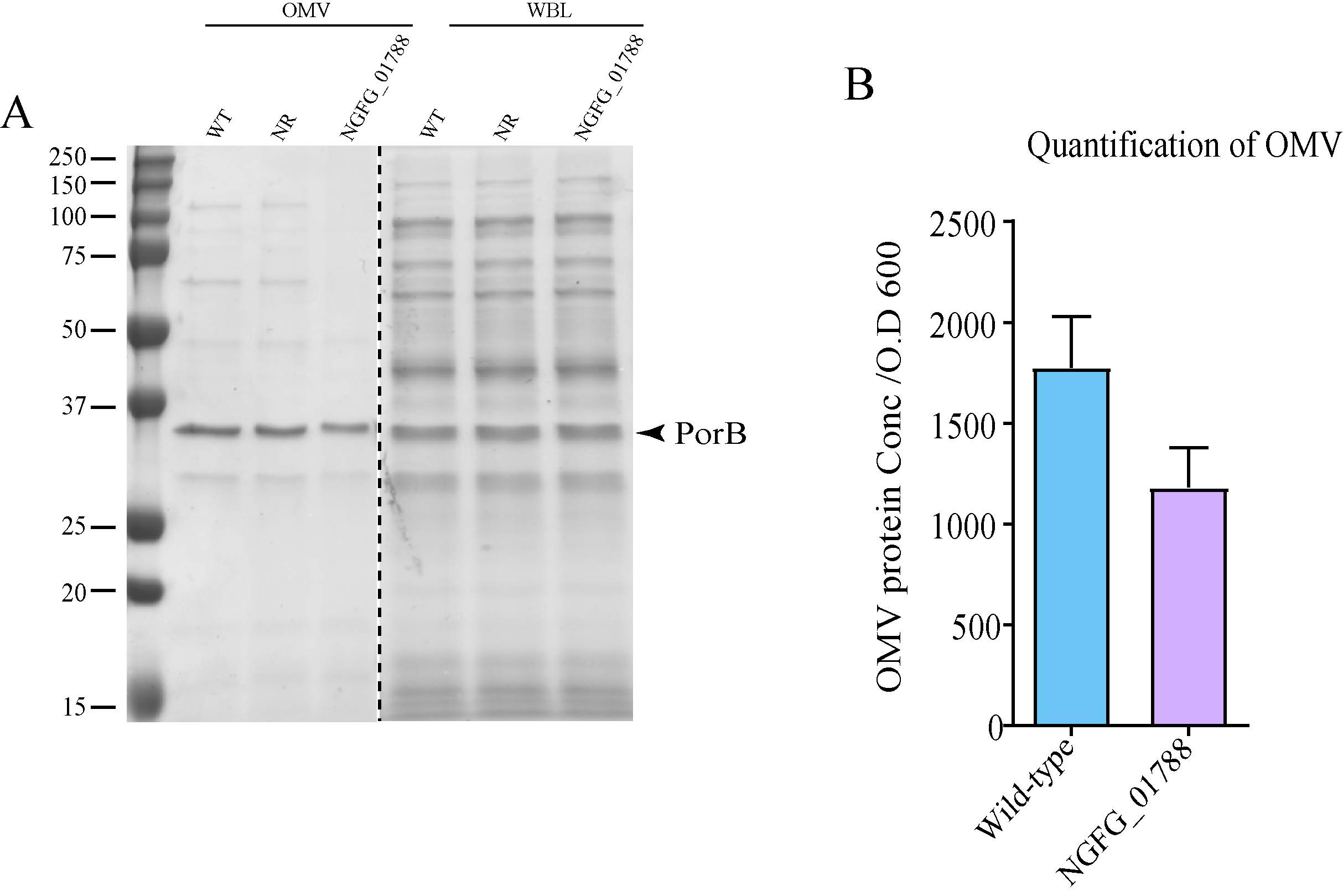

Supplement: S2 Fig — (A) Purified OMVs and whole bacterial lysates (WBL) from equal numbers of wild type (WT) and ΔNGFG_01788 deletion mutant were stained with coomassie after gel-electrophoresis. PorB is indicated based on immune blot analysis. NR indicates not-relevant lane. (B) Total protein content of purified OMVs from WT and ΔNGFG_01788 deletion mutant was determined by the BCA assay relative to bacterial numbers (OD 600 nm). Mean and SD from three independent experiments. (TIF) [file ppat.1006945.s002.tif]

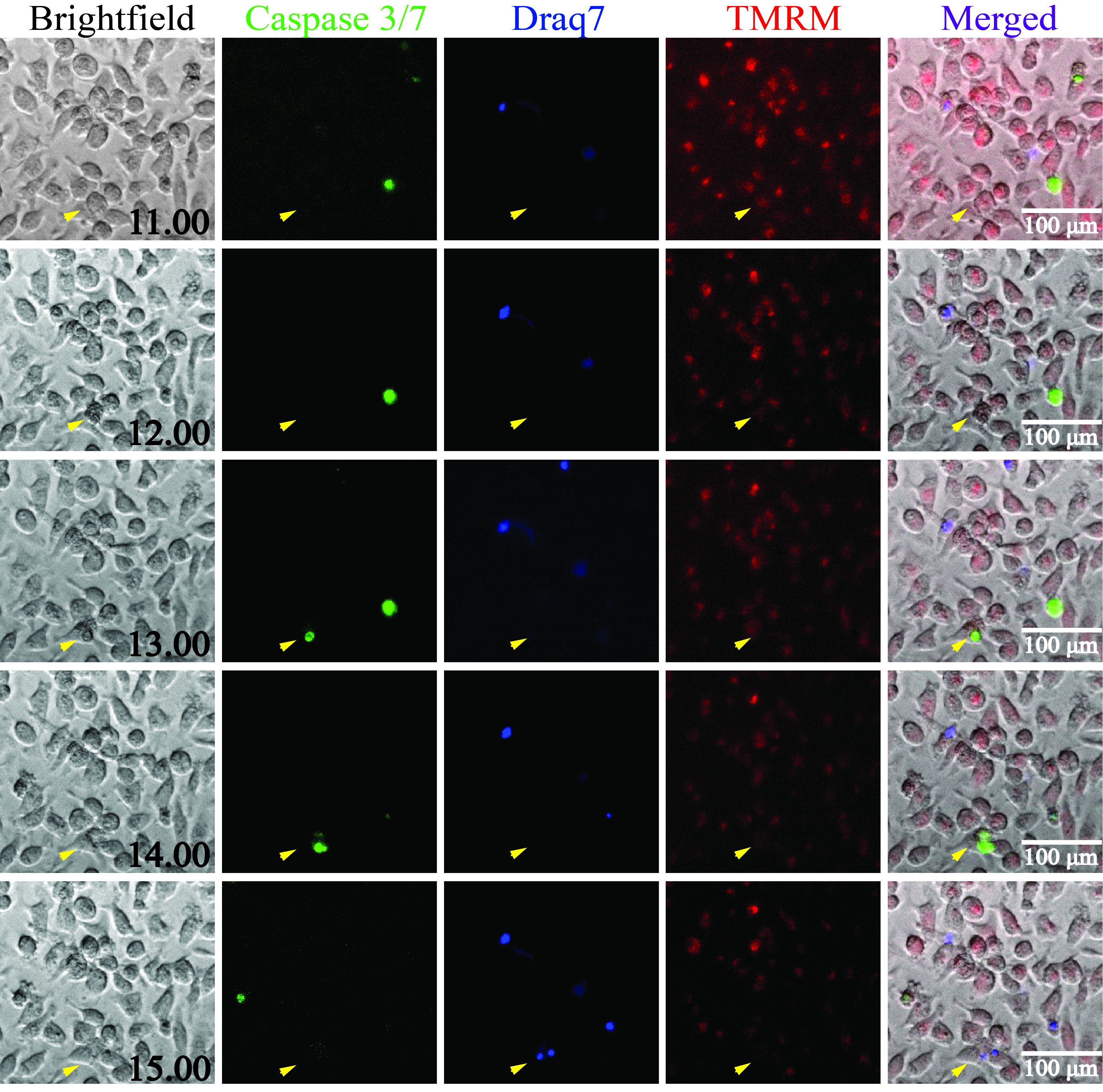

Supplement: S3 Fig — BMDMs were labelled with TMRM (red), exposed to OMVs and incubated with caspase-3/7 specific fluorogenic substrate (green) and Draq7 (blue). Time-lapse images are shown from indicated time frames. Arrow indicates a macrophage that sequentially lost TMRM signal, activates caspase-3/7 and stained positive for Draq7. Scale bar = 100 μm. (TIF) [file ppat.1006945.s003.tif]

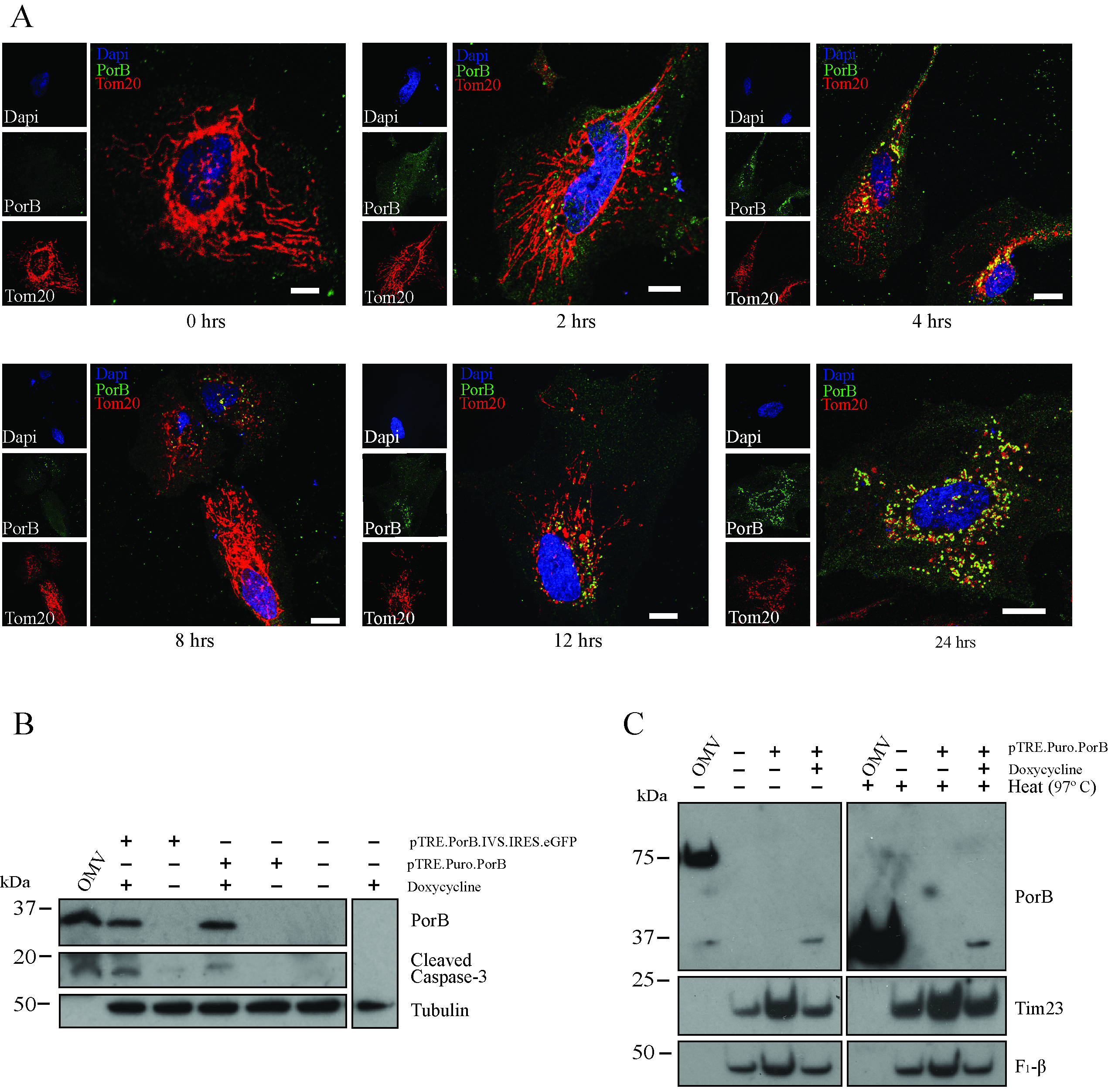

Supplement: S4 Fig — Tet-On advanced Hela cells were transiently transfected with pTRE-Tight response plasmids (pTRE.PorB.IVS.IRES.eGFP and pTRE.Puro.PorB). (A) Doxycycline dependent ectopically expressed PorB (green) colocalized with Tom20 (red) in a time dependent manner, causing the loss of mitochondrial network. (B) Doxycycline induced expression of PorB caused cleavage of caspase-3 (17 kDa) as detected by immunoblot analysis. Tubulin staining is shown as a loading control. (C) Semi-native gel electrophoresis shows heat sensitive PorB complex formation in OMVs but monomeric PorB in HeLa cells after doxycycline induction. Tim23 and F1-β are shown as loading controls. (TIF) [file ppat.1006945.s004.tif]
